# Implementing a patient-centred and evidence-based intervention to reduce BEnzodiazepine and sedative-hypnotic use to improve patient SAFEty and quality of care (BE-SAFE): study protocol of a cluster randomised controlled trial

**DOI:** 10.1136/bmjopen-2026-123804

**Published:** 2026-10-12

**Authors:** Maria-Lisa Kleinert, Sarah Atighetchi, Julia Isabel Beck, Lucy Bolt, Odile Stalder, Rita Romskaug, Enrico Callegari, Laura Fernández Maldonado, Ramon Miralles Basseda, Martha Kaznowski, Adam Wichniak, Vagioula Tsoutsi, Pinelopi Kollia, Marie De Saint-Hubert, Anne Spinewine, Nicolas Rodondi, Carole Elodie Aubert

**Affiliations:** 1 Department of General Internal Medicine, Inselspital, Bern University Hospital, University of Bern, Bern, Switzerland; 2 Institute of Primary Healthcare (BIHAM), University of Bern, Bern, Switzerland; 3 Department of Clinical Research, University of Bern, Bern, Switzerland; 4 Department of Geriatric Medicine, Oslo University Hospital, Oslo, Norway; 5 Department of Old Age Psychiatry, Østfold Hospital Trust, Grålum, Norway; 6 Fundació Salut i Envelliment, Université autonome de Barcelone, Barcelona, Spain; 7 Department of Geriatric Medicine, Hospital Germans Trias i Pujol, Université Autonome de Barcelone, Barcelona, Spain; 8 Third Psychiatric Department and Sleep Medicine Centre, Institute of Psychiatry and Neurology, Warsaw, Poland; 9 Sleep Research Unit, First Department of Psychiatry, Eginition Hospital, Medical School, National & Kapodistrian University of Athens, Athens, Greece; 10 Institute of Health and Society (IRSS), Université catholique de Louvain, Brussels, Belgium; 11 Department of Geriatric Medicine, CHU UCL Namur, Yvoir, Belgium; 12 Louvain Drug Research Institute, Clinical Pharmacy & Pharmacoepidemiology Research Group, UCLouvain, Brussels, Belgium; 13 Pharmacy Department, CHU UCL Namur, Yvoir, Belgium

**Keywords:** Benzodiazepines, Deprescriptions, Aged, Decision Making, Shared, Quality of Health Care, Patient Safety

## Abstract

**Introduction:**

The use of benzodiazepine receptor agonists (BZRA) is associated with increased risks of falls, fractures, hospitalisations and mortality, particularly in older adults, causing substantial patient harm and healthcare costs across Europe. Despite well-established safety concerns and limited effectiveness for sleep problems, BZRA remain widely prescribed. Although previous interventions, including those initiated in hospital settings, reduced BZRA use under study conditions, evidence on patient-relevant outcomes is limited and implementation in real-world European healthcare settings remains scarce. The BE-SAFE (Implementing a patient-centred and evidence-based intervention to reduce BEnzodiazepine and sedative-hypnotic use to improve patient SAFEty and quality of care) trial evaluates whether a theory-based, hospital-initiated, deprescribing intervention increases BZRA discontinuation without compromising sleep quality in older adults treated for sleep problems, compared with usual care.

**Methods and analysis:**

BE-SAFE is a multicentre, parallel group, cluster-randomised controlled trial conducted in inpatient and outpatient hospital settings across six European countries (Belgium, Greece, Norway, Poland, Spain and Switzerland). The trial assesses superiority for BZRA discontinuation and non-inferiority for sleep quality. Recruitment started in December 2024. Patients aged ≥65 years who have used BZRA for sleep problems on average ≥3 times per week for the preceding 3 months are eligible. Clusters are defined at the hospital physician level and are randomised 1:1 to usual care or the multicomponent, theory-based deprescribing intervention.

The intervention includes senior-physician endorsement; BZRA deprescribing training (including shared decision-making); a structured deprescribing algorithm; self-monitoring; patient and caregiver educational materials (including cognitive behavioural therapy for insomnia); and structured communication with community healthcare professionals.

The co-primary outcomes are BZRA discontinuation and sleep quality at 12 months. Secondary outcomes include BZRA discontinuation or dose reduction, withdrawal symptoms and sleep quality, health-related quality of life, standardised daily BZRA dose and use of substitute sleep medication; adverse effects (falls, fractures, delirium, hospitalisations and mortality). The target sample size is 470 participants across 62 clusters.

**Ethics and dissemination:**

Ethics approval was obtained from all participating centres: CHU UCL Namur, site Godinne (approval number: NUB0392024000049 – 111/2024, Belgium); CHU UCL Namur, site St. Elisabeth (approval number: NUB0392024000049 – 19/2024, Belgium); CHU UCL Namur, site Dinant (approval number: NUB0392024000049 – 5/2025, Belgium); Bioethics Committee of the Institute of Psychiatry and Neurology (approval number: 10/2024, Poland); Regional Committee for Medical and Health Research Ethics (approval number: REK sør-øst C - 722845, Norway); Ethics Committee of Eginition Hospital, Laikon Hospital, Sotiria Hospital and Ippokratio Hospital (approval numbers respectively: 1217/02-08-2024, 606/03-09-2024, 3532/10-02-2026 and 65/16-07-2024, Greece); the Commission on Ethics in Animal and Human Experimentation (CEEAH) of the Universitat Autònoma de Barcelona (approval number: 6221, Spain); Ethics Committee of Germans Trias i Pujol Hospital of Badalona (approval number PI-24-206, Spain); the Ethical Committee of the Canton of Bern (approval number: BASEC2024-00702, Switzerland). Results will be disseminated through peer-reviewed journals and presentations at scientific conferences.

**Trial registration number:**

ClinicalTrials.gov (NCT06584513); Swiss National Clinical Trials Portal (HumRes65897 | SNCTP000006133 | BASEC2024-00702).

**STRENGTHS AND LIMITATIONS OF THIS STUDY**

- Multinational, cluster-randomised controlled trial evaluating a multicomponent theory-based intervention, with the cluster design reducing contamination that would be expected with individual patient randomisation.
- The trial is explicitly designed to address both effectiveness and clinical consequences by combining a superiority outcome (benzodiazepine receptor agonist discontinuation) with a non-inferiority outcome (sleep quality) at 12 months.
- Broad eligibility criteria, including ambulatory and hospitalised patients living in the community or in nursing homes.
- As in all cluster-randomised trials, there is a potential risk of selection bias; this is minimised by blinded screening and enrolment by the research team before cluster physicians are involved, along with central monitoring of baseline characteristics.
- Blinded outcome assessment at follow-ups mitigates detection bias in a pragmatic trial where participants and physicians cannot be fully blinded to allocation.

## Introduction

 Benzodiazepine receptor agonists (BZRA), including benzodiazepines and z-drugs, are widely used for sleep problems in older adults despite clear evidence of limited benefit and substantial harm. Despite long-standing safety concerns and international clinical guidelines discouraging long-term use in older adults,^1–3^ BZRA prescribing remains common across Europe, with marked variations between countries.^4^ Among older adults, 15%–30% use BZRA, with up to 87% of prescriptions issued for sleep problems.^5–7^ BZRA use is associated with clinically relevant adverse effects such as falls, fractures, hospitalisations, dementia and mortality,^8–13^ thereby threatening patient safety. This applies especially to older adults as they are more susceptible to adverse effects due to changes in pharmacokinetics and pharmacodynamics associated with age and to the frequent presence of polypharmacy and comorbidities.^14 15^ The resulting health and economic burden is substantial, with benzodiazepine-associated falls alone costing an estimated €1.8 billion annually in hospital care in the European Union.^16^

When prescribed for sleep problems, the benefits of BZRA are modest and short-lived.^17^ Hence, guidelines recommend Cognitive Behavioural Therapy for Insomnia (CBT-I) as first-line treatment for chronic insomnia in adults.^1^ Pharmacological therapy should be reserved for short-term use (≤4 weeks) and only be considered if CBT-I is ineffective or unavailable.^1^ Nevertheless, discontinuation of BZRA is challenging due to patient-level, healthcare professional (HCP)-level and system-level barriers. Although over 42% of older adults are willing to stop BZRA following a physician’s recommendation, this willingness is often outweighed by beliefs in effectiveness and dependence.^18^ Despite awareness of risks, HCPs face structural barriers such as limited time, training and institutional support, underscoring the need for structured, theory-based interventions.^19 20^

Interventions targeting both HCPs and patients may be more effective in BZRA discontinuation, but existing trials have important limitations and limited scalability.^21–23^ The EMPOWER (Eliminating Medications Through Patient Ownership of End Results) and D-PRESCRIBE (Developing Pharmacist-led Research to Educate and Sensitize Community Residents to the Inappropriate Prescriptions Burden in the Elderly) trials used educational interventions with tapering support in pharmacies and were successful in reducing BZRA use in Canada but did not assess clinical outcomes or patient-reported outcome measures (PROMs) and excluded patients with dementia. Furthermore, their transferability to European healthcare systems is questionable.^21 23^ More recently, the Sleepwell trial tested a direct-to-patient, community-based intervention without structured HCP involvement or shared decision-making, limiting its applicability to multimorbid hospitalised populations.^24^ While deprescribing interventions may increase BZRA discontinuation, evidence for effects on patient safety, sleep quality and health-related quality of life remains of low certainty, with substantial heterogeneity across interventions.^25^ This likely reflects differences in context and implementation, as deprescribing is a longitudinal process rather than a one-time decision.

Hospitalisation may represent an opportunity to initiate BZRA deprescribing, as medication review is routinely performed.^26–28^ Evidence suggests that hospital-initiated deprescribing is feasible and may improve outcomes, with increased discontinuation at discharge and potential reductions in short-term mortality.^29 30^ However, implementation is challenged by competing priorities and transitions of care, and evidence from European healthcare systems remains limited.^31 32^ Qualitative work showed that hospital-initiated BZRA deprescribing unfolds along complex care trajectories spanning inpatient and outpatient settings. Key bottlenecks occur at shared decision-making, interprofessional communication and follow-up across transitions of care.^33^ Together, these findings highlight the need for pragmatic, scalable interventions embedded within routine care pathways in European healthcare systems.

The BE-SAFE (“Implementing a patient-centred and evidence-based intervention to reduce BEnzodiazepine and sedative-hypnotic use to improve patient SAFEty and quality of care”) trial examines whether a multicomponent, patient-centred deprescribing intervention can increase BZRA discontinuation without compromising sleep quality in older adults treated for sleep problems, compared with usual care. Beyond these co-primary outcomes assessed at 12 months, BE-SAFE evaluates patient-centred and safety outcomes complemented by a theory-based process evaluation to support adaptation and scale-up across heterogeneous European healthcare systems.

## Methods and analysis

### General study context, design and setting

The present study is part of the BE-SAFE project, a Horizon Europe initiative that aims to improve patient safety by developing, testing and implementing a patient-centred, evidence-based approach to reduce BZRA use in older adults. The development phase included the creation of evidence-based guidelines, identification of barriers and enablers, characterisation of care trajectories, and theory-informed development of the BE-SAFE intervention, all of which are described elsewhere.^18–20 25 33–35^ The BE-SAFE trial is a multicentre, parallel group, cluster-randomised controlled trial conducted in inpatient and outpatient hospital settings across 12 participating study sites in six European countries (Belgium, Greece, Norway, Poland, Spain and Switzerland), representing diverse healthcare systems. Participating sites and clinical departments were selected based on the findings of the preceding evaluation phase, which assessed the local care context and identified suitable settings for implementation. Eligible departments must have physicians interested in deprescribing BZRA in real clinical settings. Recruitment is restricted to departments with a relevant older patient population, such as general internal medicine, geriatrics and psychiatry.

The overall study design, including recruitment, cluster randomisation and follow-up procedures, is shown in figure 1.

**Figure 1:**
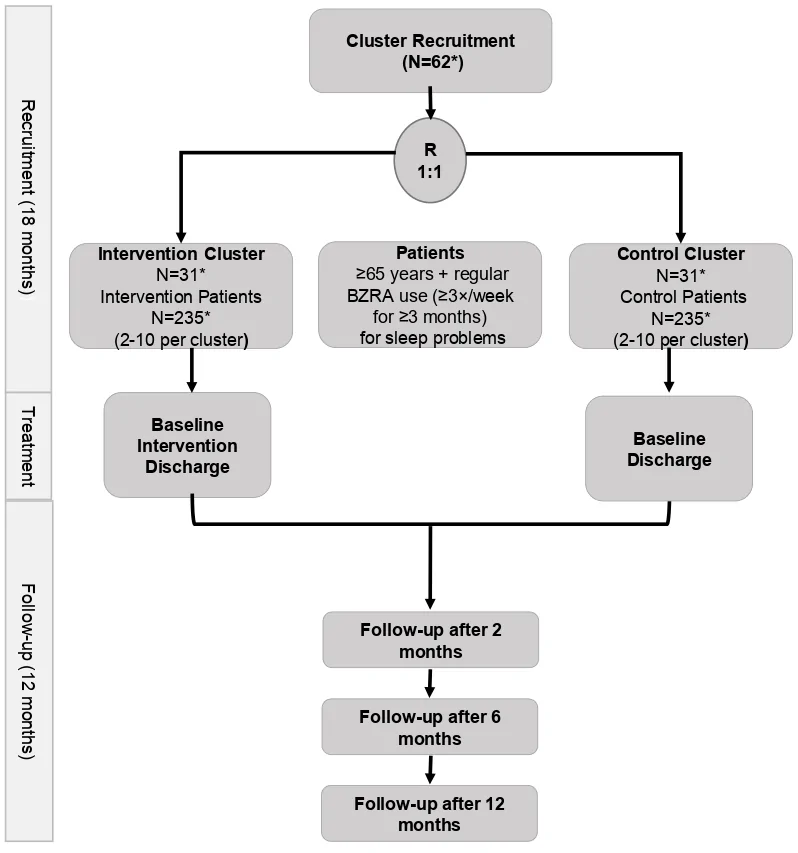
Study flowchart (*planned numbers). BZRA, benzodiazepine receptor agonists.

The trial sponsor is the Bern University Hospital, Inselspital, Bern, Switzerland.

A process evaluation is conducted alongside the trial and described in a separate protocol article. Patients are followed up at 2 (M02), 6 (M06) and 12 (M12) months; information may be provided by proxy persons. The key characteristics of the study population, intervention, control and outcomes are summarised in table 1.

**Table 1:** Population, intervention, control and outcomes

Population	Cluster level: **Chief resident physicians**, working in participating inpatient wards or outpatient clinics, expected to remain at the site for ≥**4 consecutive weeks** or until approximately 8 eligible patients per cluster have been recruited. Patient level: Consecutive **older adults** aged ≥65 years using **BZRA** on average ≥**3 times per week for at least the last 3 months** prior to study invitation, prescribed for **sleep problems**.
Intervention	**Patient-centred deprescribing intervention** for BZRA, delivered in inpatient and outpatient hospital settings, targeting physicians, patients and caregivers. The intervention comprises senior physician endorsement, physician training in BZRA deprescribing (including shared decision-making), a structured deprescribing algorithm, patient and caregiver educational materials, and structured communication to support continuity of care after discharge. Detailed intervention components are described in table 2. All intervention materials were translated into seven languages and underwent linguistic validation and cultural adaptation.
Control	**Usual care**, with physicians allocated to the control group continuing routine clinical practice and patients receiving care as normally provided without any additional intervention.
Outcomes	**Co-Primary:** BZRA discontinuation (superiority outcome) and sleep quality at 12 months after enrolment (non-inferiority outcome). The non-inferiority outcome will only be evaluated statistically if the superiority outcome is positive. **Secondary:** BZRA discontinuation or dose reduction, withdrawal symptoms and sleep quality at 2 and 6 months; health-related quality of life, standardised daily BZRA dose and use of substitute sleep medication at 2, 6 and 12 months; adverse effects of BZRA within 12 months including falls, fall-related fractures, delirium, hospitalisations and all-cause mortality.

BZRA, benzodiazepine receptor agonists.

**Table 2:** Detailed description of BE-SAFE intervention components^35^

Component	Target	Brief description
Senior physician endorsement	Physicians	A senior physician introduces the intervention in the department and emphasises to intervention cluster physicians that BZRA deprescribing in adults ≥65 years treated for sleep problems is a departmental priority, encourages completion of the training, and facilitates access to physician materials and patient brochures. The senior physician is not a cluster and is not randomised.
Training modules (videos)	Physicians	Three short modules covering: (1) rationale for BZRA deprescribing, time feasibility, CBT-I and use of the deprescribing algorithm; (2) principles and practical application of SDM; and (3) clinical case vignettes illustrating SDM and different deprescribing decisions (total duration 32 min).
Self-monitoring	Physicians	During the first 2 weeks after training, physicians are encouraged to practise with three patients they are comfortable with and reflect on performance and improvement strategies.
Deprescribing algorithm	Physicians	A stepwise, SDM-based algorithm guiding BZRA deprescribing, including clinical triggers, tapering guidance, non-pharmacological alternatives, management of patient barriers, expected benefits and risks, and links to supporting resources.
Patient/caregiver materials (brochures and videos)	Patients/caregivers	Educational brochures: “You may be at risk”—explains risks and adverse effects of long-term BZRA use in older adults* “Sleep soundly, sleep safely”—outlines non-pharmacological sleep strategies Caregiver brochure—provides practical guidance for caregivers supporting patients during deprescribing These materials are complemented by three short, animated videos that reinforce key messages and demonstrate practical sleep-management tools.
Communication tool	Patients/caregivers and HCPs	Structured communication forms are provided to patients/caregivers and other HCPs involved in the patient’s care (eg, GPs, rehabilitation physicians, nursing home staff), summarising discussions, patient values and preferences, decisions taken, tapering plans if applicable and follow-up recommendations, to support continuity of care across settings.

* The brochure “You may be at risk” was adapted from the EMPOWER trial. A Belgian adaptation of this brochure informed the version used in this study and was further refined for the participating countries, with both shared and country-specific modifications.

BE-SAFE, Implementing a patient-centred and evidence-based intervention to reduce BEnzodiazepine and sedative-hypnotic use to improve patient SAFEty and quality of care; BZRA, benzodiazepine receptor agonists; CBT-I, Cognitive Behavioural Therapy for Insomnia; GP, general practitioner; HCP, healthcare professionals; SDM, shared decision making.

This study protocol follows the Standard Protocol Items: Recommendations for Interventional Trials guidelines.^36^ The completed SPIRIT (Standard Protocol Items: Recommendations for Interventional Trials) 2025 checklist is provided in online supplemental file 1.

### Cluster definition

A cluster is defined by the chief resident physician working in one of the included settings who is responsible for prescribing medication to the included patients during their hospital care or outpatient appointment at the time of enrolment. The specific level of physician responsibility is defined at each participating site according to the local organisation of care. Subsequent involvement of other physicians according to routine clinical workflows does not alter cluster allocation.

Potentially eligible clusters are systematically identified and screened at each site by the research team. Participation is voluntary. Eligible clusters are recruited from inpatient or outpatient hospital settings. Patients remain within the same cluster until the intervention is delivered, and clusters are closed when the physician leaves the ward or outpatient clinic or at completion of the recruitment period. Physicians are expected to remain in their position for a period sufficient to reach an estimated mean cluster size (n=8 patients), typically at least four consecutive weeks. The 4-week minimum is intended to ensure sufficient opportunity for patient recruitment and does not require the BZRA tapering process to be initiated or completed during the physician’s period of participation, as the intervention may include a deprescribing plan to be implemented after discharge or in collaboration with the general practitioner (GP).

Multiple cluster physicians may work within the same department or ward while being responsible for distinct patient cohorts and may therefore be randomised to different trial arms. They are asked at inclusion not to speak about the study and its intervention with colleagues. Clusters are excluded if the ward or clinic has an existing structured medication review programme specifically targeting BZRA discontinuation, if the physician or clinical unit is participating in another interventional study aimed at optimising medication therapy, or if the physician has previously participated as a cluster in the BE-SAFE trial. Enrolment of clusters is distributed over the complete recruitment period. No country-specific quotas were applied.

### Patient inclusion criteria

Eligible patients are identified through consecutive screening and enrolled from participating clusters at each site. To maximise inclusiveness and generalisability, eligibility criteria are kept to a minimum. Adults aged ≥65 years are eligible if they have used BZRA on average ≥3 times per week during the preceding 3 months for sleep problems, as reported by the patient or an informal caregiver. Patients unable to provide informed consent, including those with dementia, are eligible if a proxy provides consent and agrees to actively participate as an informal caregiver.

### Patient exclusion criteria

Patients are excluded if continued BZRA use is clinically indicated or if withdrawal is considered unsafe. This includes the following conditions:

- Use of BZRA for alcohol withdrawal or in the context of substance use disorder.
- Rapid eye movement (REM) sleep behaviour disorder.
- Severe non-REM-related parasomnias with risk of harm to self or others or with high frequency or social embarrassment.
- Epilepsy (all forms).
- Current active diagnosis of severe general or specific anxiety disorder, psychotic disorder with or without antipsychotic medication, severe depression with or without major anxiety symptoms, bipolar disorder with or without major anxiety symptoms.
- Acute suicidal ideation.

Patients are also excluded if they are currently undergoing a physician-supported BZRA tapering process, are planned for admission to palliative care within 24 hours or have an estimated life expectancy of less than 12 months.

### Patient screening, informed consent and enrolment

Patients are identified through consecutive screening by the blinded local research team to minimise selection bias. Initial screening consists of verifying basic eligibility criteria, specifically age ≥65 years and BZRA use. These checks are performed before physician involvement and before provision of study information and formal informed consent. Patients meeting these initial criteria are subsequently assessed for all remaining inclusion and exclusion criteria, and written informed consent is obtained within 30 days of the initial screening. The baseline assessment is completed within a further 30 days after informed consent. Patients are considered enrolled at the time of informed consent signature, and time of follow-up starts at baseline assessment. Depending on the clinical pathway, these steps may take place during a single hospital stay, during an outpatient consultation, or across several outpatient visits. See online supplemental figure 1 for more details on the patient pathway.

### Randomisation

Physicians (cluster-level) are randomly assigned 1:1 to either the control or intervention group. Randomisation is stratified by country. The random allocation sequence is generated using a computer-based randomisation module implemented in REDCap (Research Electronic Data Capture). The allocation sequence is concealed within the system and is not accessible to investigators prior to assignment.

### Blinding procedure

This study is partially blinded, implemented as follows:

#### Screening and enrolment

Patient screening, enrolment and baseline data collection are performed by members of the local research team who are certified in accordance with Good Clinical Practice (GCP) and are blinded to allocation, to minimise selection bias. Patient eligibility is assessed using health records and, where necessary, additional clinical information; written informed consent is obtained prior to any study procedures and documented in accordance with local standards. All patients receive identical study information. Blinded staff perform these procedures before the cluster physician is informed of the patient’s inclusion and before intervention delivery (see online supplemental figure 1). These staff members are not involved in clinical care and thus do not collaborate with cluster physicians.

#### Patients

Patients are not explicitly informed of group allocation at enrolment; however, due to the nature of the intervention, sustained patient blinding is not feasible.

#### Physicians

At the cluster level, blinding is not feasible. Control-group physicians provide usual care and do not receive any particular training, whereas intervention-group physicians receive the intervention; all physicians receive study information and sign a non-disclosure agreement, committing not to reveal their allocation or provide training materials to other colleagues.

#### Outcome assessment

Patient-level outcomes are assessed by trained research team members blinded to intervention group allocation.

### Intervention

The BE-SAFE intervention is a theory-based, multicomponent intervention designed to enhance BZRA deprescribing initiated in hospital settings in older adults. It was developed within the BE-SAFE project following a systematic, behaviour change–based approach. The development of the physician-targeted components has been described in detail elsewhere.^35^

The intervention is inherently patient-centred and allows for flexibility in its application according to individual patient preferences and clinical judgement. Deprescribing decisions, including whether to initiate, defer or not pursue BZRA dose reduction, are made jointly by the patient and the physician. Variations in the timing, pace and extent of dose reduction (including gradual tapering or no reduction) are expected and reflect the pragmatic nature of the intervention.

Patient-facing components were developed iteratively within the BE-SAFE project with input from the Patient Partnership Advisory Council, who played a central role in reviewing, assessing and refining intervention materials to enhance patient relevance, acceptability and feasibility. The intervention targets key implementation barriers to BZRA deprescribing and comprises several interrelated components targeting physicians, patients and care transitions, with flexibility for local adaptation (table 2).

Physicians allocated to the intervention group are informed of patient inclusion only after completion of baseline data collection by blinded staff in order to minimise bias during baseline assessment. They are asked to deliver the intervention within 5 days (see online supplemental figure 1). Depending on the setting and workflow, tapering may be initiated during the same or a subsequent encounter, including during hospitalisation, after discharge or at a subsequent outpatient visit. During hospitalisation, the cluster physician delivers the intervention and determines the exact timing of intervention delivery. Deprescribing decisions are jointly agreed between the patient and cluster physician using the deprescribing algorithm. When care is transferred, the communication tool conveys the decision and tapering plan to other healthcare professionals, including the GP, to support continuity of care.

### Control group

Physicians allocated to the control group are asked to continue practice as usual. Patients in the control group receive care as usual.

### Follow-up

Patients are followed up at M02, M06 and M12 after enrolment, with assessments conducted within the prespecified time windows (±5 days). Follow-up calls are conducted with the trial participant; if the participant cannot be reached or is unable to complete the interview, a proxy person (family member or informal caregiver) is contacted where available. If required to complete missing information, the participant’s GP is contacted. For patients who die during follow-up, the cause of death is ascertained from available medical records, death certificates and autopsy reports, where available.

Standardised contact procedures, including repeated contact attempts, are used to promote retention. Participants remain in the study regardless of deprescribing decisions and are followed up according to the intention-to-treat principle; reasons for loss to follow-up are documented.

### Outcomes

#### Assessment of co-primary outcomes

The BE-SAFE cluster randomised controlled trial (RCT) has two co-primary patient-level outcomes, both assessed at M12. The first co-primary outcome is discontinuation of BZRA use, defined as self-/informal caregiver-reported intake of a maximum of one tablet within the previous 14 days. Where feasible, self-/proxy-reported BZRA discontinuation is verified using healthcare records, GP reports or other relevant sources to enhance data accuracy.

The second co-primary outcome is sleep quality at M12, assessed using the Insomnia Severity Index (ISI).^37 38^ The ISI was used in previous trials on insomnia RCTs.^39^ It is a valid outcome measure for CBT-I and preferred to other commonly used scales.^40^ Sleep quality is considered co-primary because successful BZRA discontinuation should not occur at the expense of clinically meaningful deterioration in sleep. The two co-primary outcomes are therefore assessed sequentially using a gatekeeping strategy.

#### Assessment of secondary outcomes

Secondary and safety outcomes, the instruments used for their assessment and the timing of assessments are summarised in table 3.

**Table 3:** Outcomes, measurements/instruments and times of assessment

Outcome	Measurement/instrument	M02	M06	M12
Co-primary outcomes				
BZRA discontinuation	Self-/informal caregiver report (verification via healthcare records or GP reports where feasible)			X
Sleep quality	Insomnia Severity Index (ISI)^37 38^			X
Secondary outcomes				
BZRA discontinuation or dose reduction	Self-/informal caregiver report (verification via healthcare records or GP reports where feasible)	X	X	
Sleep quality	Insomnia Severity Index (ISI)^37 38^	X	X	
Withdrawal symptoms	Clinical Institute Withdrawal Assessment Scale (CIWA-B)^59^	X	X	
Standardised daily BZRA dose	Diazepam Equivalent Dose calculation via CBIP guidelines^60^	X	X	X
Use of substitute sleep medication	Self-/informal caregiver report (verification via healthcare records or GP reports where feasible)	X	X	X
Health-related quality of life	European Quality of Life-5 Dimensions questionnaire (EQ-5D-5L)^61^	X	X	X
Safety outcomes: Falls requiring ED visit or hospitalisation Fall-related fractures Delirium Any hospitalisations Death from any cause	Self-/informal caregiver report (verification via healthcare records or GP reports where feasible)	X	X	X

BZRA, benzodiazepine receptor agonists; CBIP, Centre Belge d'Information Pharmacothérapeutique; ED, emergency department; GP, general practitioner.

### Quality control and data protection

For each enrolled patient and physician, data are recorded in an electronic case report form (eCRF) using a dedicated electronic data capture (EDC) system (REDCap). The EDC system was activated only after successful formal testing, and all data are stored on a secure server. Responsibility for hosting the EDC system, database management and central data monitoring lies with the Department of Clinical Research of the University of Bern (DCR Bern). Access to the system is role-based and password-protected, ensuring that users can access only the data required for their study responsibilities. Data transfer is encrypted, all data entries are time-stamped and any retrospective data modifications are fully traceable through an audit trail. Regular back-ups are performed and stored securely. Data quality is ensured through a risk-based, quality-by-design approach combining real-time data validation within the eCRF, central statistical data monitoring by DCR Bern and country-specific on-site monitoring coordinated centrally. Study data are collected and stored in a coded manner. Participants are identified in the eCRF only by a unique study identifier, and personal identifying information is kept confidential and accessible only to authorised personnel.

### Study duration

Participant recruitment takes place over 18 months, with individual follow-up periods of 12 months after enrolment. At the cluster level, physicians will participate until an estimated mean target of 8 patients per cluster is reached or until they leave the ward or outpatient clinic. This target is not mandatory; clusters may enrol more or fewer patients depending on recruitment dynamics. Decisions to close clusters may also be made centrally based on overall enrolment progress.

### Sample size

The sample size is based on a sequential testing procedure of the co-primary outcomes aligned with the main objectives. Superiority of the intervention for BZRA discontinuation is tested first, followed by non-inferiority for sleep quality measured by the ISI. The trial is only considered positive if both tests are significant. Sample size requirements were calculated separately for each outcome, and the larger estimate was retained, with additional allowance for uncertainty and missing data. Calculations were performed in Stata (V.16.1; StataCorp) using methods for cluster-randomised trials with variable cluster size.

In order to achieve an overall power of at least 80%, we targeted a power of at least 90% for each of the co-primary outcomes. The type I was set to a two-sided alpha of 0.05 and one-sided alpha of 0.025 for the co-primary outcomes, respectively. There is no inflation due to multiplicity as both outcomes have to be significant. For the first co-primary outcome, the trial is powered to detect a relative increase of 150% in BZRA discontinuation, corresponding to discontinuation rates of 25% in the intervention group and 10% in the control group. These assumptions are informed by previous studies, with the EMPOWER trial^21^ used as the primary reference due to its close comparability. A conservative intraclass correlation coefficient (ICC) of 0.05 was assumed.^21 41–43^ Assuming a coefficient of variation of the cluster size of 0.75 and at least 62 clusters available, a mean cluster size of 5.97 patients and an overall sample size of 370 patients are required. A Pearson χ² test without continuity correction was used.

Non-inferiority margins (second co-primary outcome) were derived from established minimally important differences and fixed at a mean difference between intervention and control of 2.5 points on the ISI. This margin is more conservative than those used in previous non-inferiority trials^44–47^ and lies outside the 95% CI for a small effect size (analogous to the 95%–95%).^48^ An SD of 6.0 and an ICC of 0.09 were assumed.^43^ This resulted in a required sample size of 470 participants (approximately 235 per arm) across at least 62 clusters (mean cluster size 7.6 patients) based on a Student t-test. With this final sample size, the power for the first primary outcome increases to 93%, while maintaining the same assumptions. All sites have sufficient eligible physicians to support recruitment of up to 135 clusters, supported by feasibility data from the OPERAM (Optimizing Therapy to Prevent Avoidable Hospital Admissions in Multimorbid Older Patients) trial.^41 42^

### Statistical analysis

All statistical analyses, including derivation of variables and definition of analysis sets, will be prespecified in a Statistical Analysis Plan (SAP) developed at the start of enrolment and finalised before 50% of the patients were enrolled, without inspection of outcome data. Analyses will be conducted at DCR Bern in accordance with established standard operating procedures.

In the primary analysis, outcomes will be analysed according to the intention-to-treat (ITT) estimand, including all enrolled participants analysed in their randomised groups regardless of protocol deviations or cross-over. To account for the cluster-randomised design, population-averaged regression models will be fitted using generalised estimating equations with an exchangeable correlation structure and robust (sandwich) standard errors with small-sample correction.^49–51^ Statistical hypothesis testing will be restricted to the two co-primary outcomes.

The first co-primary outcome, BZRA discontinuation, will be analysed using population-averaged logistic regression, with OR as the primary effect measure. The second co-primary outcome, sleep quality assessed by the ISI, will be analysed using population-averaged linear regression with mean differences as the primary effect measure. Baseline-adjusted analyses will be performed^52^ (adjustment by age, sex, ISI and number of medications), with the adjusted analysis considered primary. For interpretation, confidence distribution of the two co-primary outcomes will additionally be presented.^53^

We will also perform a secondary per-protocol (PP) adjusted analysis to support interpretation of the primary findings.^54^ Detailed criteria for protocol adherence that might cause confounding/selection bias will be prespecified in the SAP.

In line with the main objectives of the RCT, we will use a hierarchical testing procedure for the primary analysis of the primary outcomes. First, we will test whether the experimental group is superior to the control group with regard to BZRA discontinuation (the effect will be considered significant at p<0.05). Only if superiority is established, we will test for non-inferiority with regard to symptom control (the effect will be considered significant at p<0.025). If superiority cannot be established, we will not test for non-inferiority but rather present only effect measures accompanied by CIs. Secondary outcomes will be analysed analogously to the primary outcomes but restricted to treatment policy estimand. Repeated continuous outcomes will be analysed using mixed models for repeated measures with random intercept for patient and cluster, patients are nested within cluster, and visit-specific estimates will be derived from the fitted models.

Prespecified exploratory analyses will assess intervention effect heterogeneity by introducing interaction terms into the primary analysis. These analyses will be limited to country and patient-level characteristics in the full analysis set (FAS), following the intention-to-treat principle, and will be considered exploratory, without adjustment for multiple testing. Missing data are expected to be low. For treatment policy estimand, missing data will be ignored if ≤5% and imputed based on pre-enrolment and post-enrolment data otherwise.^55^ No replacement of participants who discontinue the intervention or withdraw from the study is planned. No formal interim analyses are foreseen; safety outcomes, including hospitalisations and deaths, will be monitored descriptively throughout the trial.

### Participant burden

Given the advanced age and comorbidity of the study population, follow-up assessments may be demanding. The duration of interviews is therefore being monitored in the initial phase of recruitment, and predefined secondary outcomes may be dropped if assessments prove excessively burdensome, without affecting the primary outcomes.

### Patient and public involvement

Within the BE-SAFE project, a Patient Partnership Advisory Council, comprising older patients, informal caregivers and representatives of European patient organisations, advised on patient-relevant aspects of the intervention and study procedures.

Patient input informed the development of patient-facing components, including educational materials (brochures and videos), shared decision-making tools, the personalised tapering plan structure and the informed consent. Findings from a preparatory patient survey and qualitative interviews with patients and informal caregivers further informed the selection of patient-centred outcomes and the intervention design.^18 33^ PROMs were selected with input from the advisory council to ensure relevance and acceptability. The council also contributed to identifying recruitment barriers and feasible solutions. Patients and the public are not involved in recruitment or trial conduct. Results will be disseminated through newsletters, lay summaries and collaboration with patient organisations, with advisory council input to ensure clarity and accessibility.

### Ethics and dissemination

Ethical approval for the study protocol and all relevant trial documents has been obtained from the responsible ethics committees in all participating countries (including Switzerland, BASEC ID: 2024–00702). A full list of ethics approvals, including approval numbers and participating sites, is provided in the online supplemental file 2.

Any mayor protocol amendments require prior approval from the relevant ethics committees and are communicated to investigators, trial registries and regulatory authorities as required. The study is conducted in accordance with the Declaration of Helsinki and Good Clinical Practice guidelines.^56 57^ Informed consent was obtained from all participants prior to data collection. The standard English prototype and an example of a locally adapted, ethics-approved German consent form are provided as online supplemental file 3. Safety reporting and study oversight follow national regulatory requirements, including submission of annual safety reports and notification of study completion or termination.

The results of this trial will be disseminated through peer-reviewed publications, conference presentations and reporting in clinical trial registries. In line with open-access principles, efforts will be made to ensure transparent reporting of all findings. Where appropriate, results will also be communicated to participants and the public through plain language summaries. Responsibility for dissemination lies with the study investigators.

## Discussion

To our knowledge, this trial is the first large, multinational cluster-randomised controlled trial to evaluate a patient-centred, theory-based deprescribing intervention for BZRA in older adults across diverse European healthcare settings. We aim to address whether BZRA discontinuation can be achieved without compromising sleep quality. The inclusion of patient-centred outcomes, including PROMs, and the 12-month follow-up period further strengthen the relevance of the findings for clinical practice and policy.

The trial has several important strengths. The cluster-randomised design, with the physician as the unit of randomisation, is well suited to evaluate a multicomponent behaviour-change intervention and minimises contamination that would be expected with individual patient randomisation. Broad eligibility criteria, not excluding patients living in nursing homes or with cognitive impairment, enhance generalisability to real-world older populations most affected by BZRA overuse. Blinded screening and enrolment prior to intervention delivery, central monitoring of baseline characteristics, and blinded outcome assessment at follow-up mitigate key risks of bias in this pragmatic design.

Potential limitations should also be acknowledged. As with all cluster-randomised trials, there remains a risk of selection bias. Also, full blinding of participants and physicians is not feasible given the nature of the intervention. Finally, several outcomes rely on patient or proxy report, which may result in some misclassification and be subject to recall bias, as well as respondent fatigue due to the length and complexity of follow-up assessments; nevertheless, this approach reflects routine clinical practice and is appropriate for a pragmatic trial conducted across heterogeneous healthcare systems. In addition, variability in intervention fidelity or dose may occur given the multicomponent, behaviourally oriented nature of the intervention; a process evaluation (described in a separate protocol) will thus assess implementation and help interpret trial findings in this context.

Overall, the cluster-randomised controlled BE-SAFE trial aims to generate robust evidence on the effectiveness, safety and patient-centred impact of a multicomponent deprescribing intervention to reduce BZRA use in older adults treated for sleep problems across diverse European healthcare settings. By evaluating BZRA discontinuation alongside non-inferiority in sleep quality and a broad range of patient-centred and safety outcomes, BE-SAFE seeks to address critical evidence gaps in this field. In the longer term, the trial aims to inform the implementation of context-sensitive, patient-centred deprescribing strategies and contribute to safer, higher-value care for the growing older population in Europe.

## Current status of the BE-SAFE trial

Protocol version: Version 2.3 (13 August 2025). Recruitment for the BE-SAFE trial started in December 2024 and finished in July 2026. Follow-up is ongoing.

## Supplementary material

10.1136/bmjopen-2026-123804 online supplemental file 1

10.1136/bmjopen-2026-123804 online supplemental file 2

10.1136/bmjopen-2026-123804 online supplemental file 3

10.1136/bmjopen-2026-123804 online supplemental file 4

## References

[1] Riemann D, Espie CA, Altena E (2023). The European Insomnia Guideline: An update on the diagnosis and treatment of insomnia 2023. J Sleep Res.

[2] By the 2023 American Geriatrics Society Beers Criteria® Update Expert Panel (2023). American Geriatrics Society 2023 updated AGS Beers Criteria® for potentially inappropriate medication use in older adults. J American Geriatrics Society.

[3] O’Mahony D, Cherubini A, Guiteras AR (2023). STOPP/START criteria for potentially inappropriate prescribing in older people: version 3. Eur Geriatr Med.

[4] Organisation for Economic Co-Operation and Development (2025). Health at a glance 2025: OECD indicators.

[5] Pek EA, Remfry A, Pendrith C (2017). High Prevalence of Inappropriate Benzodiazepine and Sedative Hypnotic Prescriptions among Hospitalized Older Adults. J Hosp Med.

[6] Luta X, Bagnoud C, Lambiris M (2020). Patterns of benzodiazepine prescription among older adults in Switzerland: a cross-sectional analysis of claims data. BMJ Open.

[7] Proulx J, Hunt J (2015). Drug Use among Seniors on Public Drug Programs in Canada, 2012. Healthc Q.

[8] Woolcott JC, Richardson KJ, Wiens MO (2009). Meta-analysis of the impact of 9 medication classes on falls in elderly persons. Arch Intern Med.

[9] Parsaik AK, Mascarenhas SS, Khosh-Chashm D (2016). Mortality associated with anxiolytic and hypnotic drugs—A systematic review and meta-analysis. *Aust N Z J Psychiatry*.

[10] Donnelly K, Bracchi R, Hewitt J (2017). Benzodiazepines, Z-drugs and the risk of hip fracture: A systematic review and meta-analysis. PLoS One.

[11] Ferreira P, Ferreira AR, Barreto B (2022). Is there a link between the use of benzodiazepines and related drugs and dementia? A systematic review of reviews. Eur Geriatr Med.

[12] Lombardi N, Bettiol A, Crescioli G (2020). Risk of hospitalisation associated with benzodiazepines and z-drugs in Italy: a nationwide multicentre study in emergency departments. Intern Emerg Med.

[13] Starks SCE, Pohrt A, Halzl-Yürek F (2025). Long-term and pre-operative benzodiazepine use in older adults and risk for postoperative delirium: An additional analysis of the multicentre Biomarker Development for Postoperative Cognitive Impairment in the Elderly Study. Eur J Anaesthesiol.

[14] Sateia MJ, Buysse DJ, Krystal AD (2017). Clinical Practice Guideline for the Pharmacologic Treatment of Chronic Insomnia in Adults: An American Academy of Sleep Medicine Clinical Practice Guideline. J Clin Sleep Med.

[15] Campanelli CM (2012). American Geriatrics Society updated Beers Criteria for potentially inappropriate medication use in older adults. J Am Geriatr Soc.

[16] Panneman MJ, Goettsch WG, Kramarz P (2003). The costs of benzodiazepine-associated hospital-treated fall Injuries in the EU: a Pharmo study. Drugs Aging.

[17] Holbrook AM, Crowther R, Lotter A (2000). Meta-analysis of benzodiazepine use in the treatment of insomnia. CMAJ.

[18] Shapoval V, Hubert M, Pétein C (2025). Barriers to discontinuing benzodiazepine receptor agonists in older adults: a survey of older adults across Europe. Age Ageing.

[19] Shapoval V, Evrard P, Sibille FX (2025). Barriers to and Enablers of Supporting Deprescribing Benzodiazepines in Older Adults: A Survey of European Nonphysician Healthcare Professionals. Basic Clin Pharmacol Toxicol.

[20] Shapoval V, de Saint Hubert M, Evrard P (2025). Barriers to Deprescribing Benzodiazepines in Older Adults in a Survey of European Physicians. JAMA Netw Open.

[21] Tannenbaum C, Martin P, Tamblyn R (2014). Reduction of inappropriate benzodiazepine prescriptions among older adults through direct patient education: the EMPOWER cluster randomized trial. JAMA Intern Med.

[22] Salonoja M, Salminen M, Aarnio P (2010). One-time counselling decreases the use of benzodiazepines and related drugs among community-dwelling older persons. Age Ageing.

[23] Martin P, Tamblyn R, Benedetti A (2018). Effect of a Pharmacist-Led Educational Intervention on Inappropriate Medication Prescriptions in Older Adults: The D-PRESCRIBE Randomized Clinical Trial. JAMA.

[24] Gardner DM, Turner JP, Magalhaes S (2024). Patient Self-Guided Interventions to Reduce Sedative Use and Improve Sleep: The YAWNS NB Randomized Clinical Trial. JAMA Psychiatry.

[25] Zeraatkar D, Nagraj SK, Ling M (2025). Comparative effectiveness of interventions to facilitate deprescription of benzodiazepines and other sedative hypnotics: systematic review and meta-analysis. BMJ.

[26] Sawan M, Reeve E, Turner J (2020). A systems approach to identifying the challenges of implementing deprescribing in older adults across different health-care settings and countries: a narrative review. Expert Rev Clin Pharmacol.

[27] Sibille FX, Spinewine A, Zerah L (2022). Current practice in benzodiazepine receptor agonists deprescribing on acute geriatric wards: a cohort study. BMC Geriatr.

[28] Petrovic M, Somers A, Onder G (2016). Optimization of Geriatric Pharmacotherapy: Role of Multifaceted Cooperation in the Hospital Setting. Drugs Aging.

[29] McDonald EG, Wu PE, Rashidi B (2022). The MedSafer Study—Electronic Decision Support for Deprescribing in Hospitalized Older Adults: A Cluster Randomized Clinical Trial. JAMA Intern Med.

[30] Bellelli F, Ferrara MC, Ornago AM (2026). Benzodiazepine exposure and 6-month mortality in older adults post-hospitalization: time to consider deprescribing. J Gerontol A Biol Sci Med Sci.

[31] Neville HL, Granter C, Adibi P (2022). Interventions to reduce benzodiazepine and sedative-hypnotic drug use in acute care hospitals: A scoping review. Res Social Adm Pharm.

[32] Saeed D, Carter G, Parsons C (2022). Interventions to improve medicines optimisation in frail older patients in secondary and acute care settings: a systematic review of randomised controlled trials and non-randomised studies. Int J Clin Pharm.

[33] López-Toribio M, Dalleur O, Adda TR (2025). Mapping care trajectories for hospital-initiated benzodiazepine deprescribing in older adults: a multicentre qualitative study. BMC Health Serv Res.

[34] Agoritsas T, Kumbargere Nagraj S, Callegari E (2023). Deprescription of benzodiazepine and sedative hypnotics in insomnia disorder: a clinical practice guideline. BE-SAFE deprescription recommendations.

[35] Sibille F-X, Salbert J, Bolt L (2025). Equipping physicians for benzodiazepine receptor agonists deprescription in older adults: theory-based development of the BE-SAFE intervention. Res Social Adm Pharm.

[36] Chan A-W, Boutron I, Hopewell S (2025). SPIRIT 2025 statement: updated guideline for protocols of randomised trials. BMJ.

[37] Bastien CH, Vallières A, Morin CM (2001). Validation of the Insomnia Severity Index as an outcome measure for insomnia research. Sleep Med.

[38] Morin CM, Belleville G, Bélanger L (2011). The Insomnia Severity Index: Psychometric Indicators to Detect Insomnia Cases and Evaluate Treatment Response. Sleep.

[39] Buysse DJ, Ancoli-Israel S, Edinger JD (2006). Recommendations for a Standard Research Assessment of Insomnia. Sleep.

[40] Chen PY, Jan YW, Yang CM (2017). Are the Insomnia Severity Index and Pittsburgh Sleep Quality Index valid outcome measures for Cognitive Behavioral Therapy for Insomnia? Inquiry from the perspective of response shifts and longitudinal measurement invariance in their Chinese versions. Sleep Med.

[41] Adam L, Moutzouri E, Baumgartner C (2019). Rationale and design of OPtimising thERapy to prevent Avoidable hospital admissions in Multimorbid older people (OPERAM): a cluster randomised controlled trial. BMJ Open.

[42] Blum MR, Sallevelt B, Spinewine A (2021). Optimizing Therapy to Prevent Avoidable Hospital Admissions in Multimorbid Older Adults (OPERAM): cluster randomised controlled trial. BMJ.

[43] Health Services Research Unit UoA (2020). Empirical estimates of ICCs from changing professional practice studies.

[44] Mercier J, Ivers H, Savard J (2018). A non-inferiority randomized controlled trial comparing a home-based aerobic exercise program to a self-administered cognitive-behavioral therapy for insomnia in cancer patients. Sleep.

[45] Pinto LR, Bittencourt LRA, Treptow EC (2016). Eszopiclone versus zopiclone in the treatment of insomnia. Clinics (Sao Paulo).

[46] Blom K, Tarkian Tillgren H, Wiklund T (2015). Internet-vs. group-delivered cognitive behavior therapy for insomnia: A randomized controlled non-inferiority trial. Behav Res Ther.

[47] Garland SN, Carlson LE, Stephens AJ (2014). Mindfulness-Based Stress Reduction Compared With Cognitive Behavioral Therapy for the Treatment of Insomnia Comorbid With Cancer: A Randomized, Partially Blinded, Noninferiority Trial. JCO.

[48] U.S. Food and Drug Administration (2016). Non-*inferiority clinical trials to establish effec*tiveness.

[49] Leyrat C, Morgan KE, Leurent B (2018). Cluster randomized trials with a small number of clusters: which analyses should be used?. Int J Epidemiol.

[50] Kahan BC, Forbes G, Ali Y (2016). Increased risk of type I errors in cluster randomised trials with small or medium numbers of clusters: a review, reanalysis, and simulation study. Trials.

[51] Johnson JL, Kreidler SM, Catellier DJ (2015). Recommendations for choosing an analysis method that controls Type I error for unbalanced cluster sample designs with Gaussian outcomes. Stat Med.

[52] Yang S, Starks MA, Hernandez AF (2020). Impact of baseline covariate imbalance on bias in treatment effect estimation in cluster randomized trials: Race as an example. Contemp Clin Trials.

[53] Shakespeare TP, Gebski VJ, Veness MJ (2001). Improving interpretation of clinical studies by use of confidence levels, clinical significance curves, and risk-benefit contours. Lancet.

[54] Piaggio G, Elbourne DR, Pocock SJ (2012). Reporting of noninferiority and equivalence randomized trials: extension of the CONSORT 2010 statement. JAMA.

[55] Little RJ, D’Agostino R, Cohen ML (2012). The prevention and treatment of missing data in clinical trials. N Engl J Med.

[56] Association WM (2013). World Medical Association Declaration of Helsinki: Ethical Principles for Medical Research Involving Human Subjects. JAMA.

[57] (2016). International council for harmonisation of technical requirements for pharmaceuticals for human use. ICH E6 (R2) guideline for good clinical practice.

[58] Pétein C, Dujardin N, de Montigny M (2024). Deprescribing benzodiazepine receptor agonists in older adults: a mixed-methods study to adapt the Canadian D-PRESCRIBE intervention to the Belgian community setting. BMJ Open.

[59] Busto UE, Sykora K, Sellers EM (1989). A clinical scale to assess benzodiazepine withdrawal. J Clin Psychopharmacol.

[60] Belgian Centre for Pharmacotherapeutic Information (CBIP) (2026). Benzodiazépines – conversion des benzodiazépines et Z-drugs (dose équivalente). https://www.cbip.be/fr/chapters/11?frag=7476.

[61] (1990). EuroQol - a new facility for the measurement of health-related quality of life. Health Policy.

