# Supplementary material for: Implementing a patient-centred and evidence-based intervention to reduce BEnzodiazepine and sedative-hypnotic use to improve patient SAFEty and quality of care (BE-SAFE): study protocol of a cluster randomised controlled trial
Source: BMJ Open. 2026 Oct 12;16(10):e123804. doi: 10.1136/bmjopen-2026-123804 (PMC13662039; doi:10.1136/bmjopen-2026-123804)
Supplement: Supplementary file 1 [file bmjopen-16-10-s004.pdf]

BE-SAFE

PARTICIPANT INFORMATION SHEET

Scientific title: Implementing a patient-centred and evidence-based intervention to reduce **BE**nzodiazepine and sedative-hypnotic use to improve patient **SAFE**ty and quality of care (**BE-SAFE**): a cluster randomized controlled study

Lay title: Improving patient safety by supporting older adults in managing sleep problems **(BE-SAFE study)**

Dear Sir or Madam,

We would like to invite you to participate in our study called BE-SAFE. The BE-SAFE study aims to help older adults better manage sleep problems, with the final goal to increase patient safety. It is funded by Horizon Europe and the Swiss State Secretariat for Education, Research and Innovation and conducted in several countries across Europe. We ask you to participate in this study because you are 65 years of age or older and you regularly use sleeping pills.

Before you decide if you want to participate, we ask you to carefully read this information sheet. It helps you understand why we conduct this research project and what your participation would mean for you.

Your participation is voluntary. The following information sheet aims to support you in your decision to participate or not. If you decide to participate in the BE-SAFE study, we kindly ask you to sign the consent form for participation at the end of this document. By signing, you confirm that you read and understood the participant information sheet for participants. Please do not hesitate to ask the investigator or their representative if something is unclear.

The participant information sheet and consent form consist of four parts:

Part 1 Overview
Part 2 Detailed information
Part 3 Data protection and insurance coverage
Part 4 Consent forms

Part 1 gives you an overview of the study. In **Part 2,** you receive more in-depth information. **Part 3** encloses information on data protection and insurance coverage. By signing at the end of the document in **Part 4**, you declare that you understood the information and that you agree to participate

This study is initiated by the Insel Gruppe AG. This institution is called the sponsor. The sponsor conducts the study. The responsible person on the part of the sponsor is Prof. Dr. med. Nicolas Rodondi, Director of the Institute of Primary Health Care (BIHAM), University of Bern, and Head of Ambulatory Care, Clinic of General Internal Medicine, Inselspital, Bern, Switzerland.

Responsible for participants in Switzerland:

Name: Prof. Dr. med. Nicolas Rodondi, MAS, Director of the Institute of Primary Health Care (BIHAM), University of Bern, and Head of Ambulatory Care, Clinic of General Internal Medicine, Inselspital, Bern, Switzerland.
Address: Universitätsklinik für Allgemeine Innere Medizin Inselspital – Bern.
Phone number study staff (Monday through Friday, 8h-17h): 031 632 00 69
Email: be-safe-study@insel.ch

Part 1:

Overview

1. Why do we conduct this study?

Older adults frequently have sleep problems for which they take sleeping pills (called benzodiazepines or z-drugs). However, sleeping pills have side effects, which are more frequent with aging and can thus put a patient in danger. In this study, we want to investigate an intervention that helps older adults better manage their sleep problems by using practices other than medication. Part 2, Section 4 gives you detailed information on the scientific background of this study.

2. What happens to me if I decide to participate?

Participation lasts 12 months, during which you will have one appointment at the study centre (approx. 60 minutes) and 3-4 phone calls (approx. 40 minutes each).

If you participate, it will be randomly decided if you receive the BE-SAFE intervention or the control procedure. The BE-SAFE intervention provides additional support to help with sleep problems. The control procedure is the usual care that you are already receiving. You cannot decide in which group you participate.

For this study, we may need that your family doctor, other treating physicians or your pharmacist share your medical records with the study staff. By signing this document, you consent for doing so.

Part 2, Section 5 provides more information on the process of the study.

3. What risks and benefits are associated with study participation?

Benefits

By participating in this study, you help in the evaluation of the BE-SAFE intervention. Your participation could help ameliorate how sleep problems are treated and improve patient safety. If you receive the study intervention (BE-SAFE intervention), additional support should help you better manage your sleep problems. The knowledge we gain through this study will help other persons dealing with sleep problems as well. Participation in the study does not guarantee any benefit to the patients.

Risks

During the study, you might decide with your physician to reduce or stop your sleeping pill. If you do so, you will do it step by step. The goal is to avoid withdrawal symptoms such as worsening of sleep problems, sweating and anxiety. You will always have the choice to decide if you want to reduce or stop your sleeping pill or not.

Part 2, Section 6 provides additional information on potential risks and burden related to reducing or stopping sleeping pills.

Figure 1. Potential benefits and risks of participation.

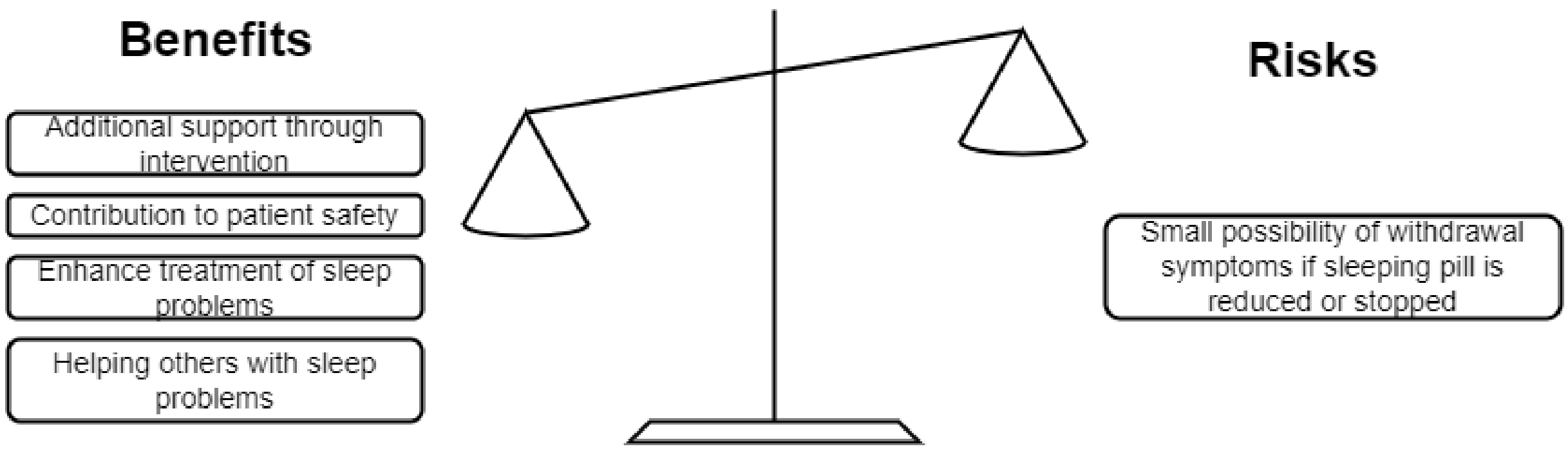

Part 2:
Detailed information

4. Scientific background

4.1 Background: Why do we conduct this study?

Sleep problems are common, especially among older adults. Sleeping pills (called benzodiazepines and z-drugs) are often prescribed to treat sleep problems. They can provoke side effects, particularly in older adults. For example, falls, fractures, hospitalisation, cognitive impairment or loss of concentration. However, there are many other ways to help with sleep problems, such as sleep hygiene or lifestyle changes. But managing sleep problems is challenging and physicians need to partner with their patients and consider their individual needs and goals.

This is why we developed the BE-SAFE intervention, in partnerships with patients who suffer from sleep problems and take sleeping pills themselves.

4.2 Structure of the study: How do we proceed?

If you decide to participate to the study, you will receive either the BE-SAFE intervention or the control procedure. The BE-SAFE intervention provides additional support to physicians and study participants to help them manage sleep problems. The control procedure is the usual care that you are already receiving. You cannot decide in which group you participate. This is decided by chance so that the study results are reliable.

4.3 Regulations on scientific research with humans

This study is conducted according to Swiss laws (Human Research Act, HRA and Federal Act on Data Protection, FADP) and international regulations. The responsible ethics committee reviewed and approved the study.

BE-SAFE is an international study. In total, we will include 470 participants across 6 European countries (Belgium, Greece, Norway, Poland, Spain, and Switzerland). Further information on this study can be found on the website of the Federal Office of Public Health at http://www.kofam.ch/ and on the study website at https://besafe-horizon.eu/.

5. Study procedures

5.1 What are your responsibilities?

If you decide to participate, the study will last 12 months for you, during which you will have one appointment at the hospital (approx. 60 minutes) at the beginning of the study, and 3 phone calls (approx. 35 minutes each) at 2, 6 and 12 months after you start to participate in the study. You might receive an additional call at month 1 (approx. 20 minutes). If you agree, we may also invite you to participate in two interviews to ask questions about your experience so far (duration approx. 30 minutes). One interview will be conducted at month 1 and another interview at month 6 after your start to participate in the study. These interviews will be organised by local researchers and the study team from Belgium. These interviews will be conducted online or by phone

and a local researcher will help you, if needed, to participate in the interview. You can still participate in the study, and refuse to participate in these interviews. We ask you to adhere to the visit schedule and to the instructions given by the study team.

Please inform the study staff if you decide to stop the study earlier (→ Section 5.3 and 5.4).

5.2 What happens during the appointments?

During the appointments, the study staff will answer any open questions and ask you questions among others about your health status, sleeping pill intake and sleep symptoms.

Figure 2. Schedule of the study.

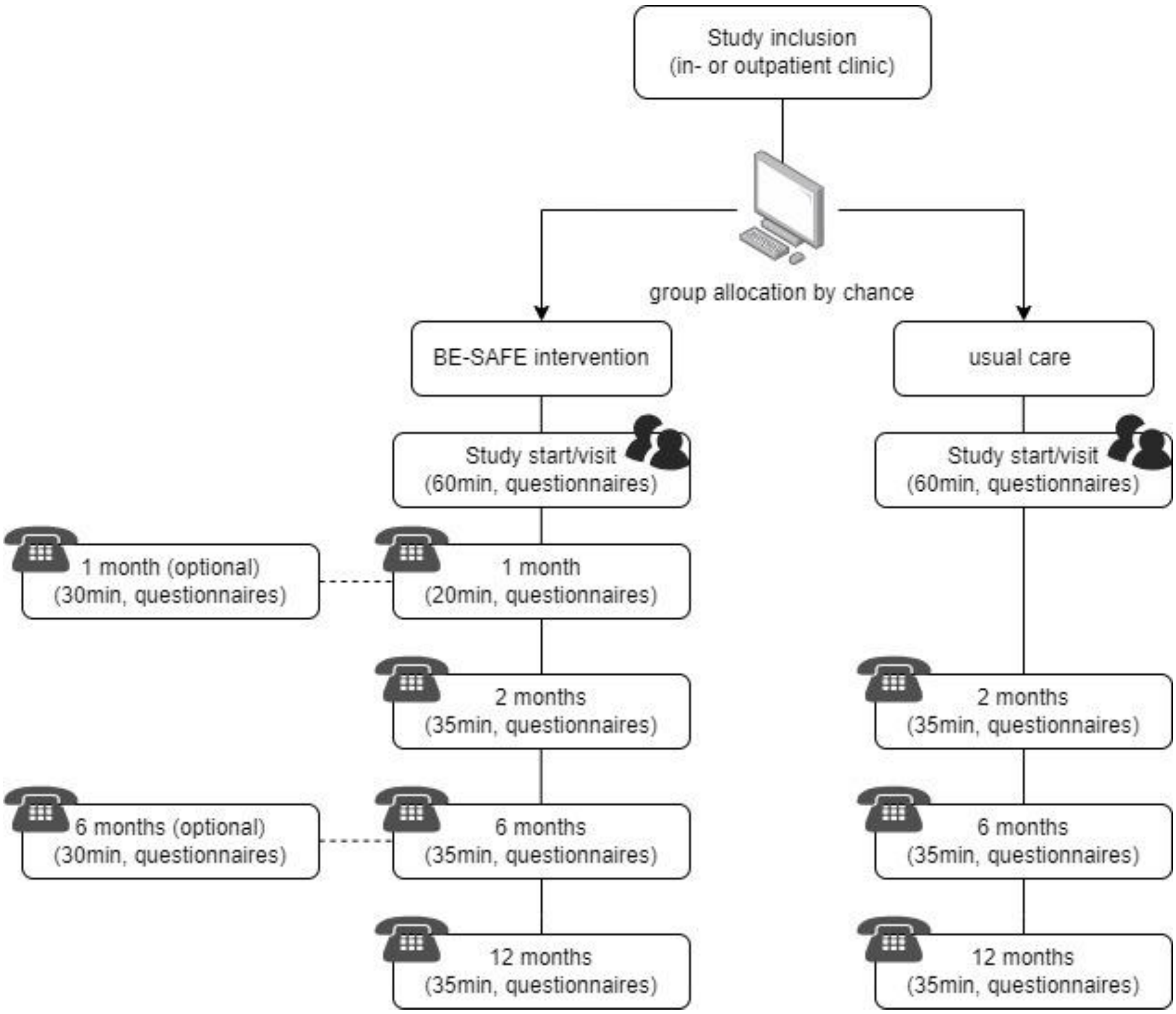

The study staff will arrange the appointments with you. You will receive an overview with all appointments listed. Please inform the study staff beforehand, if you cannot attend and have to arrange a new appointment.

5.3 When does study participation end?

Study participation lasts 12 months for each participant and ends after the last appointment. You can stop study participation at any time. You do not have to explain why you no longer want to participate. If you want to stop the study before the intended study end, please inform the study staff.

Stopping earlier will not have an effect on the further treatment you receive. You will receive your usual treatment. We will analyse data collected up to discontinuation of the study.

In certain circumstances, we will have to ask you to stop participating in the study. This can be the case if you develop another illness independently of the study, such as epilepsy, for which benzodiazepines (or sleeping pills) are a treatment of choice. However, the appointments will be completed as planned.

5.4 What happens if you decide not to participate?

If you decide not to participate in the study, you will continue to receive your usual care.

6. Risks, burden and side effects

During the study, you might decide with your hospital physician to reduce or stop your sleeping pill. If you do so, you will do it step by step. This process avoids the occurrence of withdrawal symptoms which include worsening of sleep problems, sweating, anxiety, nausea and muscle cramps. When reducing a sleeping pill step by step, the risk of withdrawal symptoms is low. Also, you will always have the choice to decide if you want to reduce or stop your sleeping pill or if you want to continue taking it.

7. Funding and compensation

This study is being funded by Horizon Europe and the Swiss State Secretariat for Education, Research and Innovation and conducted by the Insel Gruppe AG. The responsible person on the part of the sponsor is Prof. Dr. med. Nicolas Rodondi, Director of the Institute of Primary Health Care (BIHAM), University of Bern, and Head of Ambulatory Care, Clinic of General Internal Medicine, Inselspital, Bern, Switzerland.

The involved researchers do not have a direct financial benefit through conducting this study.

You will not receive any financial or other compensation if you decide to participate in this study. Participation will not lead to additional costs for you or your health insurance. We will reimburse potential travel expenses for the first appointment.

8. Study results

The overall results include data collected from all participants. This includes e.g. knowledge on how we can best support older adults in overcoming sleep problems (→ Section 4.1). These results do not affect you directly. The study staff will gladly give you an overview of the overall results at the end of study and inform you of the treatment arm you were included in.

Part 3:
Data protection and insurance coverage

9. Data protection

Your data will be protected (e.g. information on your medication or medical history). Data protection is strictly regulated by Swiss laws.

9.1 Coding of data

Every study collects data through questionnaires (e.g. medication list, medical history). These data are documented in coded form mostly electronically in large tables called data collection forms. “Coded” means that personal information (e.g. name, date of birth and place of residence) and information collected during the study are stored separately. To do this, we create a list in which every participant can be identified with a personal code. This list remains at our institution for 20 years. Nobody else other than the authorised study team can access this list.

When data are transferred – to the sponsor or other specialists conducting further research – data will always be coded and your personal data protected. This also applies if data are transferred abroad. Data will be transferred to a central site in Bern, Switzerland, for processing and storage.

9.2 Secure handling of data during the study

The sponsor, Insel Gruppe AG, and the responsible person on the part of the sponsor, Prof. Dr. med. Nicolas Rodondi, Director of the Institute of Primary Health Care (BIHAM), University of Bern, and Head of Ambulatory Care, Clinic of General Internal Medicine, Inselspital, Bern, Switzerland, are responsible for secure handling of data. The sponsor is responsible that current laws, e.g. data protection laws, are complied with. This also applies if researchers transfer data in coded form to other countries, where less strict data protection laws might apply.

In this study, the sponsor protects your data as follows:

- Researchers will collect and transfer your data electronically.
- BE-SAFE staff will save your data on a server in Switzerland.

Regardless, there remains a residual risk that strangers can access your personal data (e.g. risk of “hacking”).

For the study, it might be important that your family doctor, other treating healthcare professionals or your pharmacist share your medical records with the study staff, or that we consult your electronic records. If you unfortunately happen to die during the study time frame, we might contact your community of residence to obtain a death certificate. We might also contact your community of residence to obtain information on change of residence if we cannot reach you. By signing at the end of this document, you consent for doing so.

9.3 Secure handling of data after the study

The sponsor remains responsible for secure handling of data after completion of the study. Study documents, e.g. data collection forms, are stored for a minimum of 20 years after study completion. Any data that will be imported or exported between EU countries and Norway or Switzerland will be coded. Data for analyses will only be available in coded format. If potentially sensitive personal data will be used, it will be only during data acquisition at each participating site. BE-SAFE will follow the relevant EU guidelines, incl. Charter of Fundamental Rights of the EU, Chapter 2, Article 8: Protection of personal data; Regulation 2016/679 of the European Parliament and of the Council of 27 April 206 on the protection of natural persons with regard to the

processing of personal data on the free movement of such data, and repealing Directive 95/46/EC (General Data Protection Regulation).

After study completion ,the BE-SAFE team will publish study results in a scientific journal. For this purpose, other experts will examine the results. Thus, we will need to transfer the results in coded form. However, researchers may not use your data for other research purposes, except if you provide additional consent (→ Section 9.4).

9.4 Further use of your data in other future studies

Your data collected in this study are very important for future research and may be used for other analyses. To be able to use your data in further studies, we need you to agree on this with an additional consent. Please carefully read the informed consent form at the end of this document. Please sign the additional informed consent form, if you agree to the use of your data in future analyses. Should you refuse that we use your data for future research, you can still participate in the study.

9.5 Consent to participate in an interview

To explore participant experience of the BE-SAFE intervention, we will interview a sample of participants throughout the 6 countries at 1 and 6 months after start of the study. For these interviews, we need your additional consent. Please carefully read the informed consent form at the end of this document. Please sign the additional informed consent form, if you agree to answer the interview. Should you not consent to this, you can still participate in the study.

9.6 Inspections and quality assurance

The execution of this study can be reviewed by the responsible ethics committee or regulatory authority. Additionally, the sponsor is requested to perform reviews, to ensure the quality and results of the study. Therefore, only selected and specially trained staff can access your personal data and medical information. This means that your data is not coded for these inspections. The persons conducting the inspections are subject to confidentiality.

10. Insurance coverage

You are insured for any damage related to the study intervention. This procedure is regulated by law. The sponsor has therefore taken out insurance (*Zürich Versicherung, Eigerstrasse 2, 3007 Bern, Switzerland)*. If you believe you sustained any damage due to study participation, please contact the study staff.

Part 4:
Consent forms

This consent form includes 3 separate parts. You need to consent at least to part 1 to be able to participate to the study.

1) Consent form for participation in the study
2) Consent form for further use of personal data in future studies in coded form
3) Consent form for answer to an interview

Please read these forms carefully. Feel free to ask the study staff any questions or additional information. Your written consent is required to participate in the study.

Consent form for study participation in BE-SAFE

**BASEC-number**	xxx
**Study title**	Implementing a patient-centred and evidence-based intervention to reduce **BE**nzodiazepine and sedative-hypnotic use to improve patient **SAFE**ty and quality of care (**BE-SAFE**)
**Short title**	Improving patient safety by supporting older adults in managing sleep problems **(BE-SAFE study)**
**Sponsor**	Insel Gruppe AG
**Place of conduction**	Inselspital Bern
**Responsible Investigator at study site**	Prof. Dr. med. Nicolas Rodondi, Director of the Institute of Primary Health Care (BIHAM), University of Bern, and Head of Ambulatory

	Care, Clinic of General Internal Medicine, Inselspital, Bern, Switzerland
**Participant**<br>Full name:<br>Date of birth:	

- I received information on the study in oral and written form from the investigator or their representative signing below.
- The investigator or their representative explained the purpose, process, and risks of the study.
- My participation in the study is voluntary.
- The investigator or their representative explained standard of care treatments outside of study participation.
- I had enough time to make a decision on study participation. I will keep the written information and receive a copy of my written informed consent form.
- I can stop participating in the study at any time. I do not have to justify this decision. Despite premature study participation, I will receive further medical treatment according to standard of care. Data collected up to study discontinuation will be saved and analysed for the purpose of this study.
- The investigator or their representative can exclude me from study participation at any time if this is necessary for my health.
- I understand that my data will only be transferred in coded form. The sponsor is responsible to comply with data protection laws. All who have access to this data are bound by the Confidentiality Principles and are obliged to maintain confidentiality.
- My family doctor might be contacted during or after the end of my study participation to answer questions on their experience with the study and how it was applied.
- For controlling purposes, responsible experts of the sponsor, the ethics committee may view my uncoded data. These persons are subject to confidentiality.
- I am aware that the Inselspital Bern has taken out insurance. The insurance pays if I sustain damage directly related to study participation.

- I understand that the study personnel might contact my relatives or friends if I cannot be reached by myself.
- I understand that study personnel will contact the authorities to get information about me if they were not able to contact me.
- I understand that my family doctor, other treating physicians, other healthcare professionals and/or my pharmacist can be informed about my study participation.
- I understand that study personnel will contact my general practitioner, other physicians, other healthcare professionals (e.g., home nurse, pharmacists), the hospitals I may have been admitted to, pharmacies, and/or other medical or non-medical institutions, which provide me healthcare, to obtain my medical data.

Place and date	Full name of the participant<br><br><br>Participant's signature

Confirmation of the investigator or their **representative:** I hereby confirm that I have explained the nature, significance, and scope of the study to the participant. I assure that I will fulfil all obligations associated with this study according to local law. Should I learn of any aspects that could influence the participant's willingness to participate in the study, I will inform them immediately.

Place and date	Full name of the investigator or their representative<br><br><br>Signature of the investigator or their representative

Consent form for further use of data in coded form

This consent does not concern you in the sense of personal participation in a specific new study. "Further use" refers to retaining data beyond the time of your study participation and may be used only in coded form for further research purposes.

**BASEC-number**	xxx
**Study title**	Implementing a patient-centred and evidence-based intervention to reduce **BE**nzodiazepine and sedative-hypnotic use to improve patient **SAFE**ty and quality of care (**BE-SAFE**)
**Short title**	Improving patient safety by supporting older adults in managing sleep problems **(BE-SAFE study)**
**Participant**<br>Full name:<br>Date of birth:	

- Data collected in this study may be used in coded form for further research purposes. They will be available for future research projects for an indefinite period of time.
- I understand that my data are coded and the key is kept secure.
- Data can be analysed and stored in a database in Switzerland or abroad. Research institutes abroad must fulfil the same data protection standards as in Switzerland.
- I decide voluntarily and can revoke this decision at any time. If I withdraw, my data remain coded in the database. I only inform the investigator and do not have to provide any reason for my decision.
- Generally, data from all participants are evaluated combined. If by chance, a result appears that is very important for my health, I will be contacted. If I do not wish to be contacted, I will inform the investigator or their representative.

Place and date	Full name of the participant<br><br><br><br>Participant’s signature

Confirmation of the investigator or their representative: I hereby confirm that I have explained the nature, significance and scope of the further use of data to the participant.

Place and date	Full name of the investigator or his/her representative<br><br><br><br>Signature of the investigator or his/her representative

Consent form for answer to an interview study

I agree to participate in addition to an interview at month 1 and month 6 after start of the study.

**BASEC-number**	xxx
**Study title**	Implementing a patient-centred and evidence-based intervention to reduce **BE**nzodiazepine and sedative-hypnotic use to improve patient **SAFE**ty and quality of care (**BE-SAFE**)
**Short title**	Improving patient safety by supporting older adults in managing sleep problems **(BE-SAFE study)**
**Participant**<br>Full name:<br>Date of birth:	

- I received enough information and answers about participation in these two interviews. I understand that the interviews will be recorded to allow transcription and analysis. The transcription will be anonymised.
- I understand that, even if I agreed to participate, I might not be contacted for the interview.
- I had enough time to make a decision on participation to the interview.
- I decide voluntarily and can revoke this decision anytime. I only inform the investigator and do not have to provide any reason for my decision.
- I understand that my data are transferred to the responsible researchers in Belgium. They are liable to data protection regulations that are comparable to Swiss regulations.
- The investigators are responsible to comply with data protection laws.

Place and date	Full name of the participant

	Participant's signature

Confirmation of the investigator or their representative: I hereby confirm that I have explained the nature, significance and scope of the participation to an interview to the participant.

Place and date	Full name of the investigator or their representative<br><br>Signature of the investigator or their representative

Anfrage zur Teilnahme an medizinischer Forschung

Studientitel: Implementing a patient-centred and evidence-based intervention to reduce **BE**nzodiazepine and sedative-hypnotic use to improve patient **SAFE**ty and quality of care (**BE-SAFE**)

Laienverständlicher Titel: Patientenzentrierte Behandlungsmethode zur Senkung des Schlafmittelgebrauchs zur Verbesserung der Patientensicherheit **(BE-SAFE-Studie)**

Sehr geehrte Dame, sehr geehrter Herr

Hiermit möchten wir Sie fragen, ob Sie an unserem Forschungsprojekt BE-SAFE teilnehmen möchten. Das Ziel des Projekts ist, ältere Personen zu unterstützen, besser mit ihren Schlafproblemen umzugehen, um die Patientensicherheit zu verbessern. Die Studie wird von Horizon Europe und vom Staatssekretariat für Bildung, Forschung und Innovation finanziert und in mehreren europäischen Ländern durchgeführt. Wir fragen Sie an, ob Sie an dieser Studie teilnehmen möchten, da Sie 65 Jahre alt oder älter sind und regelmässig Schlafmittel einnehmen.

Ihre Teilnahme ist freiwillig. Die folgende **Patienteninformation** soll Ihnen bei der Entscheidung helfen, ob sie an der Studie teilnehmen möchten. Wenn Sie teilnehmen wollen, unterzeichnen Sie bitte am Ende dieses Dokumentes die **Einwilligungserklärung**. Mit Ihrer Unterschrift bestätigen Sie, dass Sie die Patienteninformation gelesen und verstanden haben. Wenn Sie etwas nicht verstehen, fragen Sie bitte die Prüfperson.

Die Patienteninformation und Einwilligungserklärung bestehen aus vier Teilen:

Teil 1 **Das Wichtigste in Kürze**
Teil 2 **Darum geht es im Detail: Informationen zur Studie**
Teil 3 **Datenschutz und Versicherungsschutz**
Teil 4 **Einwilligungserklärung**

Wenn Sie **Teil 1** lesen, dann erhalten Sie einen Überblick über die Studie. In **Teil 2** erklären wir Ihnen den ganzen Ablauf und Hintergrund der Studie im Detail. **Teil 3** enthält die Informationen zum Daten- und Versicherungsschutz. Mit Ihrer Unterschrift am Ende des Dokuments, **Teil 4**, bestätigen Sie, dass Sie alles verstanden haben und mit der Teilnahme einverstanden sind.

Sponsor der Studie ist die Insel Gruppe AG. Der Sponsor leitet eine Studie. Prof. Dr. med. Nicolas Rodondi, Chefarzt an der Universitätsklinik für Allgemeine Innere Medizin, Universitätsspital Bern (Inselspital), und Direktor vom Berner Institut für Hausarztmedizin (BIHAM), Universität Bern, ist der Vertreter des Sponsors in der Schweiz.

Im Rahmen dieser Studie ist für Sie zuständig:

Name: Prof. Dr. med. Nicolas Rodondi, Chefarzt an der Universitätsklinik für Allgemeine Innere Medizin, Universitätsspital Bern (Inselspital) & Direktor vom Berner Institut für Hausarztmedizin (BIHAM), Universität Bern

Adresse: Universitätsklinik für Allgemeine Innere Medizin Inselspital – Bern

Telefon Studienteam (Montag bis Freitag 8h-17h): 031 632 00 69

Email: be-safe-study@insel.ch

Teil 1: Das Wichtigste in Kürze

1. Warum führen wir diese Studie durch?

Ältere Person haben oft Schlafprobleme, wofür sie Schlafmittel (sogenannte Benzodiazepine und andere Sedativ-Hypnotika) einnehmen. Schlafmittel haben aber Nebenwirkungen, insbesondere bei älteren Personen, und gefährden dadurch die Patientensicherheit. In dieser Studie untersuchen wir eine Behandlungsmethode, die ältere Personen unterstützen soll, ihre Schlafprobleme anders als mit Medikamenten zu bewältigen. In Kapitel 4 erfahren Sie mehr zum wissenschaftlichen Hintergrund der Studie.

2. Was müssen Sie tun, wenn Sie teilnehmen?

Die Teilnahme an dieser Studie dauert für Sie 12 Monate. Wir werden mit Ihnen Termine für einen Studienbesuch vor Ort (Dauer ca. 60 Minuten) und 3-4 Telefongespräche (Dauer ca. 35 Minuten) vereinbaren.

Wenn Sie teilnehmen, werden Sie zufällig einer von 2 Gruppen zugeteilt: BE-SAFE-Behandlungsmethode oder Kontrollmethode. Die BE-SAFE-Behandlungsmethode bietet zusätzliche Unterstützung zur Bewältigung von Schlafproblemen. Die Kontrollmethode ist die übliche Behandlung, die Sie schon erhalten. Sie dürfen nicht frei wählen, welcher Gruppe Sie zugeteilt werden.

Im Rahmen dieser Studie kann es notwendig sein, dass Ihre Ärzt:innen (Hausarzt/Hausärztin oder andere Ärzt:innen) oder Ihr:e Apotheker:in medizinische Unterlagen dem Studienteam zusenden lassen. Mit Ihrem Unterschrift bestätigen Sie, dass Sie damit einverstanden sind.

In **Kapitel 5** erfahren Sie mehr zum Ablauf und Vorgehen der Studie.

3. Welcher Nutzen und welches Risiko sind mit der Teilnahme verbunden?

3.1 Nutzen

Mit Ihrer Teilnahme tragen Sie zur Beurteilung der BE-SAFE-Behandlungsmethode bei und helfen, die Behandlung von Schlafproblemen und somit die Patientensicherheit zu verbessern. Wenn Sie der BE-SAFE-Behandlungsmethode zugeteilt sind, dann erhalten Sie zusätzliche Unterstützung zur Bewältigung von Schlafproblemen. Die zusätzlichen Kenntnisse, die die Studie bringt, helfen anderen Patientinnen und Patienten ihre Schlafprobleme besser zu bewältigen. Die Teilnahme an der Studie bringt den Patienten keinen sicheren Nutzen.

3.2 Risiko

Möglicherweise entscheiden Sie mit Ihrem Arzt / Ihrer Ärztin im Rahmen der Studie Ihr Schlafmittel zu reduzieren oder abzusetzen. Wenn Sie sich dafür entscheiden, wird es schrittweise durchgeführt. Das Ziel ist, Entzugssymptome wie z.B. Verschlechterung von Schlafproblemen, Schwitzen oder Angst zu vermeiden. Sie dürfen immer entscheiden, ob Sie Ihr Schlafmittel reduzieren bzw. absetzen möchten oder nicht.

In **Kapitel 6** finden Sie weitere Informationen zu Risiken und Belastungen.

Abbildung 1. Mögliche Nutzen und Risiken der Teilnahme.

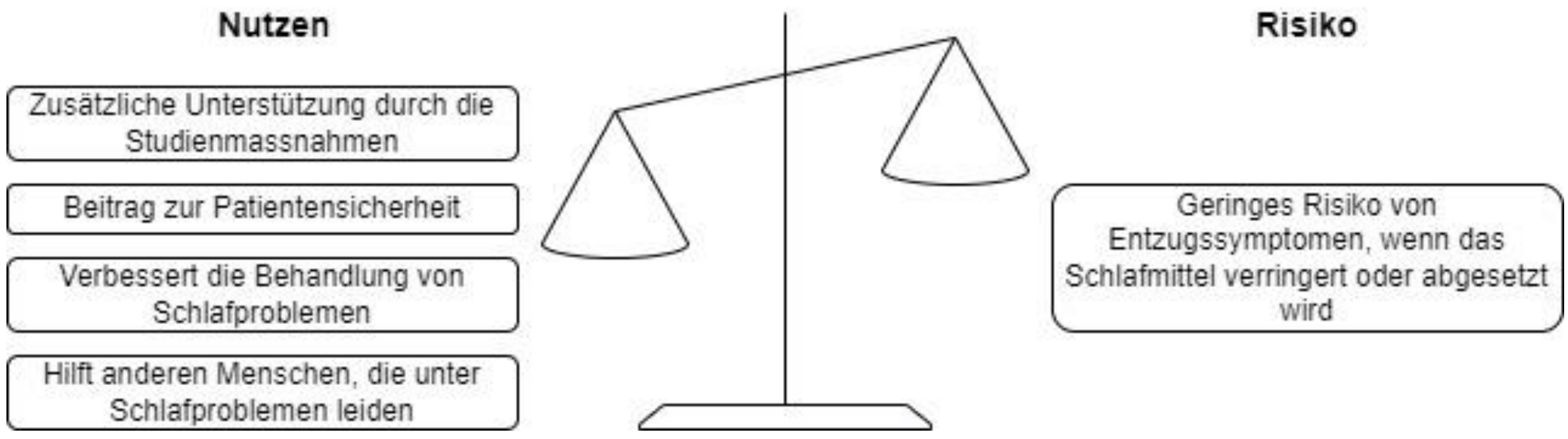

Teil 2: Darum geht es im Detail: Informationen zur Studie

4. Der wissenschaftliche Hintergrund der Studie

4.1 Hintergrund: Warum führen wir diese Studie durch?

Schlafstörungen sind weit verbreitet, insbesondere bei älteren Personen. Zur Behandlung von Schlafstörungen werden häufig Schlafmittel (sogenannte Benzodiazepine und Sedativ-Hypnotika) verschrieben. Insbesondere bei älteren Personen können diese Medikamente aber zu Nebenwirkungen führen. Dies sind zum Beispiel Stürze, Knochenbrüche, Spitalaufenthalte, Störungen der Denkleistung oder Konzentrationsstörungen. Es gibt aber viele andere Möglichkeiten, Schlafstörungen zu bewältigen, unter anderem durch Anpassungen des Lebensstils und der Schlafgewohnheiten. Schlafprobleme zu behandeln ist aber eine Herausforderung und dafür müssen Ärzt:innen mit ihren Patient:innen zusammenarbeiten und individuelle Bedürfnisse und Ziele berücksichtigen.

Aus diesem Grund haben wir die BE-SAFE-Behandlungsmethode in Partnerschaft mit Patient:innen, die an Schlafproblemen leiden und Schlafmittel einnehmen, entwickelt.

4.2 Aufbau der Studie: Wie gehen wir vor?

Wenn Sie sich entscheiden teilzunehmen, erhalten Sie entweder die BE-SAFE-Behandlungsmethode oder die Kontrollmethode. Mit der BE-SAFE-Behandlungsmethode erhalten Sie und Ihre Ärzt:innen zusätzliche Unterstützung zur Bewältigung von Schlafproblemen. Die Kontrollmethode ist die übliche Behandlung, die Sie schon erhalten. Sie dürfen nicht entscheiden, welcher Gruppe Sie zugeteilt werden. Es wird zufällig entschieden, damit die Resultate der Studie zuverlässig sind.

4.3 Regelungen zur wissenschaftlichen Forschung mit Menschen

Wir machen diese Studie so, wie es die Gesetze in der Schweiz vorschreiben (Humanforschungsgesetz, Datenschutzgesetze). Ausserdem beachten wir alle international anerkannten Richtlinien. Die zuständige Ethikkommission hat die Studie geprüft und bewilligt.

BE-SAFE ist eine internationale Studie. Wir werden insgesamt 470 Teilnehmende in der Schweiz und in 5 weiteren europäischen Ländern (Belgien, Griechenland, Norwegen, Polen, Spanien) einschliessen.

Eine Beschreibung dieser Studie finden Sie auch auf der Internetseite des Bundesamtes für Gesundheit unter www.kofam.ch und der Internetseite der Studie https://besafe-horizon.eu/de/home.

5. Ablauf der Studie

5.1 Was müssen Sie tun, wenn Sie an der Studie teilnehmen?

Die Teilnahme an der Studie ist freiwillig und dauert 12 Monate, während denen ein Termin vor Ort am Inselspital (ca. 60 Minuten) und 3 Telefongespräche (jeweils ca. 35 Minuten) 2, 6 und 12 Monate nach Studienbeginn stattfinden. Möglicherweise erhalten Sie einen zusätzlichen Telefonanruf nach einem Monat (Dauer ca. 20 Minuten). Wenn Sie einverstanden sind, laden wir Sie noch zu 2 Interviews nach 1 und nach 6 Monaten zur Befragung über Ihre Erfahrungen mit der Studie (Dauer ca. 30 Minuten) ein. Diese Interviews werden durch das Forschungsteam von Bern mit Kolleg:innen aus Belgien organisiert. Die Interviews werden online oder telefonisch durchgeführt und eine Person des Forschungsteams von Bern unterstützt Sie bei der Teilnahme, wenn nötig. Sie dürfen an der BE-SAFE-Studie trotzdem teilnehmen, selbst wenn Sie bei den Interviews nicht mitmachen möchten. Sie sollten sich an den Ablaufplan halten und auch an die Vorgaben, die Ihre Prüfperson macht.

Bitte informieren Sie Ihre Prüfperson, wenn Sie nicht mehr an der Studie teilnehmen wollen. (→ **Kapitel 5.3 und 5.4**)

5.2 Was passiert bei den Terminen?

Während den Terminen werden Ihre Fragen beantwortet und wir stellen Ihnen Fragen unter anderem über Ihren Gesundheitszustand, Ihre Einnahme von Schlafmitteln und Ihre Schlafsymptome.

Abbildung 2. Ablauf der Studie

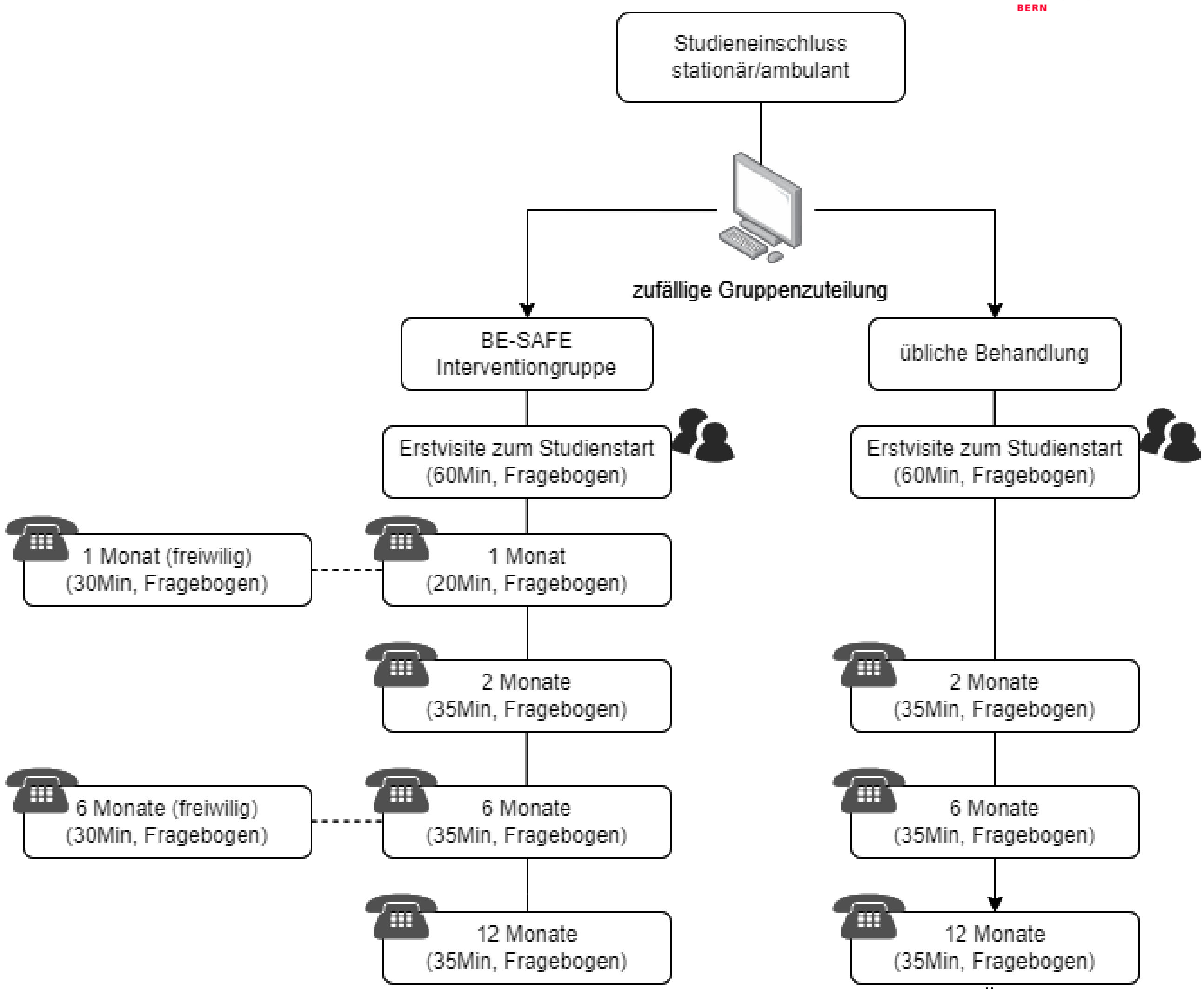

Wir vereinbaren die Termine gemeinsam mit Ihnen. Sie erhalten einen genauen Überblick über die vereinbarten Termine. Wir bitten Sie, uns schnell zu informieren, wenn Sie einmal einen Termin aus wichtigen Gründen verschieben müssen, damit wir einen neuen Termin organisieren können.

5.3 Wann endet die Teilnahme an der Studie?

Für Sie dauert die Teilnahme 12 Monate und endet nach dem letzten Termin. Sie können Ihre Teilnahme jederzeit auch früher abbrechen (→ **Kapitel 5.4**). Sie müssen nicht erklären, warum Sie nicht mehr teilnehmen möchten. Wenn Sie Ihre Teilnahme frühzeitig beenden möchten, informieren Sie bitte Ihre Prüfperson.

Auch wenn Sie Ihre Teilnahme vorzeitig beenden, behandeln und betreuen wir Sie weiter medizinisch so gut wie möglich nach den aktuellen Standards. Wenn Ihre Teilnahme vorzeitig endet, werden wir die bis dahin erhobenen Daten noch für die Studie auswerten.

Es kann sein, dass wir Sie bitten müssen, die Studie frühzeitig zu beenden. Das ist beispielsweise der Fall, wenn Sie unabhängig der Studie eine andere Erkrankung entwickeln, wie z.B. Epilepsie, wofür eine Behandlung mit Benzodiazepinen (oder Schlafmitteln) empfohlen wird. Die Termine werden jedoch wie geplant abgeschlossen.

5.4 Was passiert, wenn Sie nicht teilnehmen möchten?

Auch wenn Sie nicht an dieser Studie teilnehmen, behandeln und betreuen wir Sie medizinisch bestmöglich nach den aktuellen Standards.

6. Risiken, Belastungen und Nebenwirkungen

Möglicherweise entscheiden Sie sich mit Ihrem Arzt / Ihrer Ärztin, im Rahmen der Studie ihr Schlafmittel zu reduzieren oder abzusetzen. In diesem Fall wird es schrittweise durchgeführt, um Entzugssymptome wie Verschlechterung von Schlafproblemen, Schwitzen, Angst, Übelkeit oder Muskelkrämpfe zu vermeiden. Mit einer schrittweisen Reduktion ist das Risiko für Entzugssymptome tief. Sie dürfen auch immer entscheiden, ob Sie Ihr Schlafmittel reduzieren bzw. absetzen möchten oder nicht.

7. Finanzierung und Entschädigung

Diese Studie wird durch Horizon Europe und das Staatssekretariat für Bildung, Forschung und Innovation finanziert und durch die Insel Gruppe AG (Sponsor) durchgeführt. Prof. Dr. med. Nicolas Rodondi, Chefarzt an der Universitätsklinik für Allgemeine Innere Medizin, Universitätsspital Bern (Inselspital), und Direktor vom Berner Institut für Hausarztmedizin (BIHAM), Universität Bern, ist der Vertreter des Sponsors.

Die beteiligten Forschenden haben keinen unmittelbaren finanziellen Vorteil an der Durchführung dieser Studie.

Sie erhalten keine finanzielle oder andere Entschädigung für Ihre Teilnahme. Durch die Teilnahme an der Studie entstehen keine zusätzlichen Kosten für Sie oder für Ihre Krankenkasse. Reisekosten, die durch die Teilnahme entstehen, werden wir Ihnen zurückerstatten.

8. Ergebnisse aus der Studie

Die Gesamtergebnisse der Studie bestehen aus den Daten von allen Teilnehmenden. Dazu gehört zum Beispiel, dass wir mehr darüber wissen werden, wie wir am besten Patient:innen unterstützen können, Schlafprobleme zu bewältigen (→ **Kapitel 4.1**). Diese Ergebnisse betreffen Sie und Ihre Gesundheit nicht direkt. Wenn Sie das wünschen, gibt Ihre Prüfperson Ihnen am Ende der Studie gerne eine Zusammenfassung der Gesamtergebnisse der Studie und informiert Sie, welche Behandlungsmethode Sie erhalten haben.

Teil 3: Datenschutz und Versicherungsschutz

9. Schutz von Daten

Wir schützen Ihre Daten (z.B. Angaben zu ihren Medikamenten oder weitere medizinische Informationen aus Ihrer Krankengeschichte). Zum Schutz von Daten und Proben gibt es in der Schweiz strenge gesetzliche Regelungen.

9.1 Verschlüsselung von Daten

Bei jeder Studie entstehen Daten aus den Fragebögen (z.B. Medikamentenlisten, bekannte Erkrankungen). Diese Daten werden dokumentiert. Das passiert meist elektronisch in grossen Tabellen, den sogenannten «Datenerhebungsbögen». Alle Daten werden verschlüsselt dokumentiert. «Verschlüsselt» heisst, dass persönliche Informationen wie Name, Geburtsdatum oder Wohnort *getrennt* von den Untersuchungsergebnissen aufbewahrt werden. Dazu gibt es eine Liste, die jede Person mit einem eindeutigen Code identifiziert. Diese Liste bleibt für die Dauer von 20 Jahren an der Institution. Niemand ausser das Forschungsteam bekommt diese Liste.

Wenn wir Daten weitergeben – an den Sponsor oder an Fachpersonen, die weitere Untersuchungen machen – dann sind die Daten immer verschlüsselt und Ihre persönlichen Daten sind geschützt. Das gilt auch, wenn die Daten ins Ausland weitergegeben werden.

9.2 Sicherer Umgang mit den Daten während der Studie

Der Sponsor (Insel Gruppe AG) und seine Vertretung, Prof. Dr. med. Nicolas Rodondi, Chefarzt an der Universitätsklinik für Allgemeine Innere Medizin, Universitätsspital Bern (Inselspital), und Direktor vom Berner Institut für Hausarztmedizin (BIHAM), Universität Bern, sind für den sicheren Umgang mit Ihren Daten und Proben aus dieser Studie verantwortlich. Sie sind dafür verantwortlich, dass die geltenden Gesetze, z.B. die Datenschutzgesetze, eingehalten werden. Dies gilt auch, wenn (verschlüsselte) Daten oder Proben für Untersuchungen in Länder verschickt werden, wo die Datenschutzgesetze weniger gut sind.

So schützt der Sponsor dieser Studie Ihre Daten: In dieser Studie werden Ihre Daten elektronisch erfasst und übermittelt. Das BE-SAFE-Forschungsteam speichert Ihre Daten auf einem Server in der Schweiz. Trotzdem gibt es immer ein gewisses Restrisiko, dass fremde Personen auf Ihre persönlichen Daten zugreifen (z.B. Risiko von „Hacking").

Für diese Studie kann es wichtig sein, dass Ihr Hausarzt / Ihre Hausärztin, andere Ärzt:innen, anderes Gesundheitspersonal oder Ihr Apotheker / Ihre Apothekerin uns Ihre Krankengeschichte schickt oder dass wir Ihre elektronische Krankengeschichte prüfen. Würden Sie leider während der Studie versterben, dann würden wir Ihre Gemeinde kontaktieren, um einen Totenschein zu erhalten. Falls wir Sie nicht erreichen können, wenden wir uns ggf. auch an Ihre Wohngemeinde, um Informationen zum Wohnortwechsel einzuholen. Mit der Einwilligung am Schluss des Dokuments erlauben Sie das.

9.3 Sicherer Umgang mit Daten und Proben nach der Studie

Der Sponsor bleibt auch nach Ende der Studie verantwortlich für den sicheren Umgang mit Ihren Daten und Proben. Das Gesetz schreibt vor, dass alle Studiendokumente, z.B. die Datenerhebungsbögen, für mindestens 20 Jahre aufbewahrt werden. Alle Daten, die zwischen EU-Ländern und Norwegen oder der Schweiz importiert oder exportiert werden, werden verschlüsselt. Falls empfindliche Daten gebraucht werden, dann passiert das nur während der Datensammlung am Inselspital.

Nach Abschluss einer Studie werden die Ergebnisse meist in wissenschaftlichen Zeitschriften veröffentlicht. Dazu werden die Ergebnisse durch andere Fachpersonen begutachtet. Ihre verschlüsselten Daten müssen dabei an diese Fachpersonen weitergeleitet werden. Die Daten

dürfen allerdings nicht für neue Forschungszwecke weiterverwendet werden. Dafür braucht es Ihre separate Einwilligung (→ **Kapitel 9.4**).

9.4 Weiterverwendung Ihrer Daten in anderen, zukünftigen Studien

Ihre Daten aus dieser Studie sind für die zukünftige Forschung sehr wichtig. Daten, die für diese Studie nicht schon vollständig verwendet wurden, können möglicherweise für andere Studien weiterverwendet werden. Das ist freiwillig. Bitte lesen Sie die zusätzliche Einwilligungserklärung am Schluss des Dokuments genau durch. Unterschreiben Sie bitte die Einwilligung, wenn Sie mit Ihren Daten weitere Forschung in der Zukunft unterstützen möchten. Auch wenn Sie dem nicht zustimmen, können Sie trotzdem an der Studie teilnehmen.

9.5 Zusage zur Teilnahme an einem Interview

Um die Erfahrungen mit der BE-SAFE-Behandlungsmethode zu evaluieren, wird eine Auswahl von Teilnehmer:innen aus den 6 Ländern 1 und 6 Monate nach Studienbeginn während einem Interview telefonisch befragt. Für dieses Interview müssen Sie zusätzlich zusagen. Bitte lesen Sie die Einverständniserklärung am Ende dieses Dokuments sorgfältig durch. Bitte unterzeichnen Sie die zusätzliche Einverständniserklärung, wenn Sie mit der Beantwortung des Interviews einverstanden sind. Sollten Sie damit nicht einverstanden sein, können Sie trotzdem an der Studie teilnehmen.

9.6 Einsichtsrechte bei Kontrollen

Die Durchführung dieser Studie kann überprüft werden. Die Überprüfung geschieht durch Behörden wie die zuständige Ethikkommission, die Zulassungsbehörde *Swissmedic* oder auch durch ausländische Zulassungsbehörden. Auch der Sponsor muss solche Überprüfungen machen, damit die Qualität dieser Studie und die Ergebnisse gesichert sind.

Dafür erhalten wenige, speziell dafür ausgebildete Personen Einblick in Ihre persönlichen Daten und in Ihre Krankengeschichte. Für diese Überprüfung sind die Daten also *nicht* verschlüsselt. Die Personen, die Ihre unverschlüsselten Daten sehen, unterliegen der Schweigepflicht.

10. Versicherungsschutz

Sie sind versichert, wenn Sie durch die Studie – also durch die Behandlungsmethode – einen Schaden erleiden. Das Vorgehen ist gesetzlich geregelt. Dafür hat der Sponsor eine Versicherung abgeschlossen bei Zürich Versicherung, Eigerstrasse 2, 3007 Bern. Wenn Sie meinen, dass Sie einen Schaden durch die Studie erlitten haben, wenden Sie sich bitte an Ihre Prüfperson.

Teil 4: Einwilligungserklärungen

Diese Einwilligung besteht aus 3 unabhängigen Einwilligungserklärungen. Sie müssen mindestens Teil 1 unterschreiben, um an der Studie teilnehmen zu dürfen.

1) Einwilligungserklärung zur Teilnahme an der Studie
2) Einwilligungserklärung für die Weiterverwendung von Daten aus dieser Studie in verschlüsselter Form
3) Einwilligungserklärung zur Teilnahme an einem Interview

Bitte lesen Sie diese Formulare sorgfältig durch. Bitte fragen Sie uns, wenn Sie etwas nicht verstehen oder wenn Sie noch etwas wissen möchten. Für die Teilnahme ist Ihre schriftliche Einwilligung notwendig.

Einwilligungserklärung zur Teilnahme an der Studie

**BASEC-Nummer:**	2024-00702
**Titel der Studie**	Implementing a patient-centred and evidence-based intervention to reduce **BE**nzodiazepine and sedative-hypnotic use to improve patient **SAFE**ty and quality of care (**BE-SAFE**)
**Laienverständlicher Titel**	Patientenzentrierte Behandlungsmethode zur Reduktion von Schlafmitteln zur Verbesserung der Patientensicherheit **(BE-SAFE-Studie)**
**Verantwortliche Institution** (Sponsor mit Adresse)	Insel Gruppe AG<br>Freiburgstrasse<br>3010 Bern
**Ort der Durchführung**	Inselspital Bern
**Prüfperson am Studienort**	Prof. Dr. med. Nicolas Rodondi<br>Universitätsklinik für Allgemeine Innere Medizin<br>Universitätsspital Bern (Inselspital)
**Teilnehmerin/ Teilnehmer:**<br>Name und Vorname in Druckbuchstaben:<br>Geburtsdatum:	

- Ich habe mündlich und schriftlich Informationen über die Studie bekommen, und zwar von der Prüfperson, die unten unterschreibt.
- Die Prüfperson hat mir den Zweck, den Ablauf und die Risiken der Studie erklärt.
- Ich nehme freiwillig an der Studie teil.
- Die Prüfperson hat mir erklärt, welche möglichen Standardbehandlungen es ausserhalb der Studie gibt.
- Ich hatte genügend Zeit, um diese Entscheidung zu treffen. Ich behalte die schriftliche Information und erhalte eine Kopie meiner schriftlichen Einwilligungserklärung.
- Ich kann jederzeit meine Teilnahme beenden. Ich muss nicht erklären, warum. Auch wenn ich die Teilnahme beende, bekomme ich weiter meine medizinische Behandlung. Die Daten, die bis dahin gesammelt wurden, bleiben gespeichert und werden im Rahmen der Studie ausgewertet.
- Wenn es besser für meine Gesundheit ist, kann mich die Prüfperson jederzeit von der Studie ausschliessen.
- Ich habe verstanden, dass meine Daten nur in verschlüsselter Form weitergegeben / ins Ausland gesendet werden. Der Sponsor sorgt dafür, dass der Datenschutz nach Schweizer Standard eingehalten wird.

- Meine Hausärztin / mein Hausarzt wird eventuell während oder nach Ende meiner Teilnahme an der Studie kontaktiert, um einige Fragen über ihre / seine Erfahrung mit der Studie und wie die Behandlungsmethode angewendet wurde zu beantworten.
- Die zuständigen Fachpersonen des Sponsors, der Ethikkommission und/oder der Arzneimittelbehörde *Swissmedic* dürfen meine unverschlüsselten Daten zur Kontrolle einsehen. Alle diese Personen unterstehen der Schweigepflicht.
- Ich weiss, dass das Inselspital Bern eine Versicherung abgeschlossen hat. Diese Versicherung bezahlt, wenn ich einen Schaden erleide – aber nur, wenn der Schaden direkt mit der Studie zusammenhängt.
- Ich verstehe, dass das Forschungsteam Angehörige oder Kolleg:innen kontaktieren darf, wenn ich nicht erreichbar bin.
- Ich verstehe, dass das Forschungsteam die Behörden kontaktieren wird, um Information über mich einzuholen, wenn ich nicht erreichbar bin.
- Ich verstehe, dass meine Hausärztin / mein Hausarzt, meine anderen Ärzt:innen, meine anderen pflegenden Personen und/oder mein:e Apothekerin / mein Apotheker informiert werden können, dass ich an der Studie teilnehme.
- Ich verstehe, dass das Forschungsteam meine Hausärztin / meinen Hausarzt, andere Ärzt:innen, anderes Gesundheitspersonal (z.B. Pflegeheim, Apotheker:innen), das Spital, in dem ich stationär behandelt wurde, und/oder andere medizinische oder nicht-medizinische Institutionen, die mich behandeln, kontaktieren wird, um meine medizinische Daten zu erhalten.

Ort, Datum	Name und Vorname Teilnehmerin / Teilnehmer<br>in Druckbuchstaben<br><br>Unterschrift Teilnehmerin / Teilnehmer

Bestätigung der Prüfperson: Hiermit bestätige ich, dass ich dieser Teilnehmerin / diesem Teilnehmer Art, Bedeutung und Tragweite der Studie erläutert habe. Ich versichere, alle mit dieser Studie stehenden Verpflichtungen nach Schweizer Recht zu erfüllen. Sollte ich im Verlauf der Studie von Aspekten erfahren, welche die Bereitschaft der Teilnehmerin / des Teilnehmers zur Studienteilnahme beeinflussen könnten, werde ich sie / ihn umgehend darüber informieren.

Ort, Datum	Name und Vorname der Prüfperson in Druckbuchstaben<br><br>Unterschrift der Prüfperson

INSELGRUPPE

UNIVERSITÄT BERN

Einwilligungserklärung zur Weiterverwendung von Daten in verschlüsselter Form

Diese Einwilligung betrifft Sie nicht im Sinne der persönlichen Teilnahme an einer Studie. «Weiterverwendung» meint, dass Daten über die Zeit Ihrer Studienteilnahme hinaus aufbewahrt und in verschlüsselter Form für weitere Forschung verwendet werden können.

**BASEC-Nummer:**	2024-00702
**Titel der Studie**	Implementing a patient-centred and evidence-based intervention to reduce **BE**nzodiazepine and sedative-hypnotic use to improve patient **SAFE**ty and quality of care (**BE-SAFE**)
**Laienverständlicher Titel**	Patientenzentrierte Behandlungsmethode zur Reduktion von Schlafmitteln zur Verbesserung der Patientensicherheit (**BE-SAFE-Studie**)
**Teilnehmerin/Teilnehmer:**<br>Name und Vorname in Druckbuchstaben:<br>Geburtsdatum:	

- Ich erlaube, dass meine verschlüsselten Daten aus dieser Studie für die medizinische Forschung weiterverwendet werden dürfen. Sie stehen dann für zukünftige, weitere Forschungsprojekte auf unbestimmte Zeit zur Verfügung.
- Ich habe verstanden, dass die Daten verschlüsselt sind und der Schlüssel sicher aufbewahrt wird.
- Die Daten können im In- und Ausland ausgewertet werden und in einer Datenbank hier oder im Ausland gespeichert werden. Forschungsinstitutionen im Ausland müssen dieselben Standards zum Datenschutz einhalten, wie sie in der Schweiz gelten.
- Ich entscheide freiwillig und kann diesen Entscheid zu jedem Zeitpunkt wieder zurücknehmen. Wenn ich zurücktrete, werden alle meine Daten anonymisiert. Ich informiere lediglich meine Prüfperson und muss diesen Entscheid nicht begründen.
- Normalerweise werden alle Daten zusammengefasst ausgewertet. Wenn sich zufällig ein Ergebnis zeigt, das für meine Gesundheit sehr wichtig ist, werde ich kontaktiert. Wenn ich das nicht wünsche, teile ich dies meiner Prüfperson mit.

Ort, Datum	Name und Vorname Teilnehmerin / Teilnehmer<br>in Druckbuchstaben<br><br>Unterschrift Teilnehmerin / Teilnehmer

Bestätigung der Prüfperson: Ich bestätige, dass ich der Teilnehmerin/dem Teilnehmer Art, Bedeutung und Tragweite der Weiterverwendung von Daten erläutert habe.

Ort, Datum	Name und Vorname der Prüfperson in Druckbuchstaben<br><br>Unterschrift der Prüfperson

INSELGRUPPE

UNIVERSITÄT BERN

Einwilligungserklärung zur Teilnahme an einem Interview

Ich willige ein, zusätzlich an einem Interview 1 und 6 Monate nach meinem Studienbeginn teilzunehmen.

**BASEC-Nummer:**	2024-00702
**Titel der Studie**	Implementing a patient-centred and evidence-based intervention to reduce **BE**nzodiazepine and sedative-hypnotic use to improve patient **SAFE**ty and quality of care (**BE-SAFE**)
**Laienverständlicher Titel**	Patientenzentrierte Behandlungsmethode zur Reduktion von Schlafmitteln zur Verbesserung der Patientensicherheit (**BE-SAFE-Studie**)
**Teilnehmerin/Teilnehmer:**<br>Name und Vorname in Druckbuchstaben:<br>Geburtsdatum:	

- Ich habe genügend Information und Antworten über die Teilnahme an diesen 2 Interviews erhalten. Ich verstehe, dass die Interviews aufgenommen werden, um die Transkription und Analyse zu erlauben.
- I verstehe, dass ich vielleicht nicht für ein Interview kontaktiert werde, obwohl ich zugesagt habe.
- Ich hatte genügend Zeit, um diese Entscheidung zu treffen.
- Ich nehme freiwillig an den Interviews teil und darf mich jederzeit zurückziehen. Ich muss nur die Prüfperson über meine Entscheidung informieren, ohne sie begründen zu müssen.
- Ich verstehe, dass meine Daten zu den verantwortlichen Forscher:innen in Belgien weitergeleitet werden. Sie unterliegen Datenschutzbedingungen, die mit dem schweizerischen Gesetz vergleichbar sind.
- Die Prüfperson ist für die Einhaltung des Datenschutzgesetzes verantwortlich.

Ort, Datum	Name und Vorname Teilnehmerin / Teilnehmer in Druckbuchstaben<br><br>Unterschrift Teilnehmerin / Teilnehmer

Bestätigung der Prüfperson: Ich bestätige, dass ich der Teilnehmerin/dem Teilnehmer Art, Bedeutung und Tragweite der Teilnahme an einem Interview erläutert habe.

Ort, Datum	Name und Vorname der Prüfperson in Druckbuchstaben<br><br>Unterschrift der Prüfperson
