# Supplementary material for: Implementing a patient-centred and evidence-based intervention to reduce BEnzodiazepine and sedative-hypnotic use to improve patient SAFEty and quality of care (BE-SAFE): study protocol of a cluster randomised controlled trial
Source: BMJ Open. 2026 Oct 12;16(10):e123804. doi: 10.1136/bmjopen-2026-123804 (PMC13662039; doi:10.1136/bmjopen-2026-123804)
Supplement: Supplementary file 2 [file bmjopen-2026-123804.reviewer_comments.pdf]

PEER REVIEW HISTORY

BMJ Open publishes all reviews undertaken for accepted manuscripts. Reviewers are asked to complete a checklist review form and are provided with free text boxes to elaborate on their assessment. These free text comments are reproduced below.

ARTICLE DETAILS

Title (Provisional)

Implementing a patient-centred and evidence-based intervention to reduce BEnzodiazepine and sedative-hypnotic use to improve patient SAFEty and quality of care (BE-SAFE): study protocol of a cluster randomised controlled trial

Authors

Kleinert, Maria-Lisa; Atighetchi, Sarah; Beck, Julia Isabel; Bolt, Lucy; Stalder, Odile; Romskaug, Rita; Callegari, Enrico; Fernández Maldonado, Laura; Miralles Basseda, Ramon; Kaznowski, Martha; Wichniak, Adam; Tsoutsi, Vagioula; Kollia, Pinelopi; De Saint-Hubert, Marie; Spinewine, Anne; Rodondi, Nicolas; Aubert, Carole Elodie

---

VERSION 1 - REVIEW

**Reviewer**	**1**
**Name**	**Grischott, Thomas**
**Affiliation**	**University Hospital Zurich, Institute of Primary Care**
**Date**	**04-Aug-2026**
**COI**	**None**

The authors present a protocol for an essentially well-thought-out and carefully planned international multicenter trial on deprescribing of benzodiazepines and sedatives (BZRA). I have made various comments (many of which are merely suggestions or recommendations) directly in the PDF and ask the authors to carefully consider them.

The main points are as follows:

- Selection of centres and clusters: The selection of hospitals/departments/wards is not explained at all, and the recruitment of clusters (= prescribing physicians) is unclear. For instance, how and by whom are clusters recruited? Are there country quotas? Is participation mandatory for physicians once the senior/department head/hospital director has agreed? How is potential selection bias on the levels mentioned (hospitals through to clusters) prevented or addressed?

- Study procedures & patient pathway: Please clarify the sequence of screening and enrolment. Are eligibility checks (including verification of 3-month BZRA use) conducted

prior to patient information and consent? If so, what is the timeframe between screening and actual inclusion? At what specific point are patients actually enrolled (during outpatient consultations vs. at hospital admission)? When is the intervention actually carried out (right at or shortly after admission, or at discharge; in the first or a subsequent outpatient visit)? The intervention possibly includes tapering over a period of time; when does this happen and who is responsible? Please illustrate the patient pathway through all study steps, ideally for both inpatient and outpatient settings. For instance in the inpatient setting: Who is when defined as the cluster-defining, prescribing physician when the admitting and discharging physicians are different? Does the intervention only affect discharge medication, or is potential tapering already started during the hospital stay? Or, for an outpatient with a single visit: Is consent obtained in advance before the patient visits in person, or is there enough time for everything during one visit? Authors should try to provide a more concrete description of the practical workflows, grounded in the realities of day-to-day hospital operations.

- Contamination: Are cluster-defining physicians restricted to one senior staff member per hospital/department/ward, or are physicians from the same department/ward randomized into the same study arm (which would introduce an additional level of clustering)? If not, how is contamination mitigated if multiple senior physicians are sequentially responsible for an inpatient? How does the design handle physician turnover, especially handovers between physicians from different trial arms, to prevent cross-contamination? If patients are enrolled on admission, the admitting senior physician will probably act as the cluster-defining physician, but the attending physician making subsequent medication changes and managing their stay and discharge and who actually carries out the deprescribing might not participate in the study or even define a cluster in the opposing study arm. How is this handled/addressed? How are intervention continuity and fidelity maintained during planned handovers of cluster-defining physicians or senior staff, or in cases of short-notice schedule changes?

- Local research teams: The authors mention local research teams, but their role is unclear. Please explain who recruits and who belongs to these teams in both inpatient and outpatient settings. Can local research teams "serve" clusters of both arms simultaneously? How realistic is assuming blinded local research teams when they closely collaborate with prescribing (cluster-defining) physicians who are aware of their group allocation?

- How much/far are subsequent physicians (e.g. in rehabilitation hospitals) or medical staff in nursing homes etc. involved? Are they systematically invited to continue potential tapering? This should be described in a little more detail.

- Standardization of BZRA: How are different BZRAs compared (e.g., via equivalence tables)? This is relevant since dose reduction is a secondary outcome. If BZRA 1 is replaced by BZRA 2 then how is a dose reduction defined?

- Power & sample size: If sample sizes for two co-primary outcomes are calculated separately to yield 90% power, simply taking the maximum of the two sample sizes does not guarantee 90% power to demonstrate an effect on both outcomes. The authors do not state what overall power they aim to achieve for showing effects on both outcomes (or whether they plan to just demonstrate "at least one effect", which would be an unusual interpretation of co-primary outcomes). The authors should probably re-evaluate their sample size calculation and explicitly state underlying assumptions (crucial: co-endpoints := effect in both endpoints sought, or just in at least one?). Additionally, the actual sample sizes for both endpoints should be reported separately.

- Test terminology: "Superiority" is defined differently across relevant sources and regulatory guidelines. The authors should clarify their definition/understanding and also explain their somewhat unusual choice of their significance level of alpha = 2.5%.

- Missing data: How are (e.g., intermittent) missing values handled for participants who do not drop out?

- Text/article structure: The final section of the manuscript (essentially p. 21) is repetitive, with strengths mentioned multiple times, and with a duplicate conclusion. Please shorten this as suggested in the annotated PDF, which I once again ask the authors to review.

**** The reviewer provided a marked copy with additional comments. Please contact the publisher for full details. ****

VERSION 1 - AUTHOR RESPONSE

REVIEWER

Introduction and study rationale

COMMENT 1 – REVIEWER

A BE-SAFE trial is mentioned in the abstract but not the broader(?) BE-SAFE project, and both do not appear in the main text so far. Consider briefly introducing it first.

ANSWER 1 – REVIEWER

Thank you for pointing that out. We introduced a brief description of the BE-SAFE project in our *Methods and Analysis* section:

Page 6-7, lines 156-162:

"The present study is part of the BE-SAFE ("Implementing a patient-centred and evidence-based intervention to reduce BEnzodiazepine and sedative-hypnotic use to improve patient SAFEty and quality of care") project, a Horizon Europe initiative that aims to improve patient safety by developing, testing, and implementing a patient-centred, evidence-based approach to reduce BZRA use in older adults. The development phase included the creation of

evidence-based guidelines, identification of barriers and enablers, characterisation of care trajectories, and theory-informed development of the BE-SAFE intervention, all of which are described elsewhere. (3-9)"

COMMENT 2 – REVIEWER

Selection of centres and clusters: The selection of hospitals/departments/wards is not explained at all, and the recruitment of clusters (= prescribing physicians) is unclear. For instance, how and by whom are clusters recruited? Are there country quotas? Is participation mandatory for physicians once the senior/department head/hospital director has agreed? How is potential selection bias on the levels mentioned (hospitals through to clusters) prevented or addressed?

ANSWER 2 – REVIEWER

We have clarified the selection of participating sites and clinical departments, as well as the recruitment of clusters, in the *General study context, design and setting* as well as *Cluster definition* sections. The participating hospitals and clinical departments were selected based on the evaluation phase. Eligible departments had to have physicians interested in deprescribing BZRA in real clinical settings, and to care for older adults. Physicians of those departments were systematically identified and screened by the study team; participation was voluntary and not mandatory, but physicians were strongly encouraged to participate. There were no country-specific quotas.

Page 7, lines 162-170:

"The BE-SAFE trial is a multicentre, parallel group, cluster-randomised controlled trial conducted in inpatient and outpatient hospital settings across 12 participating study sites in six European countries (Belgium, Greece, Norway, Poland, Spain, and Switzerland), representing diverse healthcare systems. Participating sites and clinical departments were selected based on the findings of the preceding evaluation phase, which assessed the local care context and identified suitable settings for implementation. Eligible departments must have physicians interested in deprescribing BZRA in real clinical settings. Recruitment is restricted to departments with a relevant older patient population, such as general internal medicine, geriatrics, and psychiatry."

Page 9-10, lines 201-218:

"Potentially eligible clusters are systematically identified and screened at each site by the research team. Participation is voluntary. Eligible clusters are recruited from inpatient or outpatient hospital settings. […] Enrolment of clusters is distributed over the complete recruitment period. No country-specific quotas were applied."

Cluster definition, physician turnover and contamination

COMMENT 3 – REVIEWER

Contamination: Are cluster-defining physicians restricted to one senior staff member per hospital/department/ward, or are physicians from the same department/ward randomized into the same study arm (which would introduce an additional level of clustering)? If not, how is contamination mitigated if multiple senior physicians are sequentially responsible for an inpatient? How does the design handle physician turnover, especially handovers between physicians from different trial arms, to prevent cross-contamination?

How does this design handle physician turnover (especially handovers between residents from different trial arms) to prevent cross-contamination? Are cluster-defining physicians restricted to senior staff? how are intervention continuity and fidelity maintained during planned or handovers of senior staff? please elaborate.

<u>ANSWER 3 – REVIEWER</u>

In BE-SAFE, the cluster is defined at the level of the individual prescribing physician, rather than at the level of the department or ward. Thus, multiple physicians working within the same department or ward may be individually randomised to different trial arms; the department or ward is not considered an additional unit of randomisation or clustering. Departments with very high-frequency physician turnover, where responsibility for patients changes daily or every few days, were considered unsuitable because a stable prescribing physician could not be reliably identified.

To avoid contamination at the prescribing physician level, the clusters are asked not to speak about the study or the intervention with their colleagues. As this is a pragmatic trial embedded in routine hospital care, subsequent prescribing decisions by other physicians cannot be completely controlled and some contamination cannot be excluded. However, subsequent physicians do not automatically assume responsibility for delivering the BE-SAFE intervention, and any subsequent medication changes remain subject to clinical judgement and the patient's previously agreed treatment plan. The potential for variability in intervention implementation across care transitions is possible and will be assessed as part of the separate process evaluation.

We clarified these aspects in the *Cluster definition* section, on page 9, lines 193-205:

"A cluster is defined by the chief resident physician working in one of the included settings who is responsible for prescribing medication to the included patients during their hospital care or outpatient appointment at the time of enrolment. The specific level of physician responsibility is defined at each participating site according to the local organisation of care. Subsequent involvement of other physicians according to routine clinical workflows does not alter cluster allocation.
Potentially eligible clusters are systematically identified and screened at each site by the research team. Participation is voluntary. Eligible clusters are recruited from inpatient or outpatient hospital settings. Patients remain within the same cluster until the intervention is delivered, and clusters are closed when the physician leaves the ward or outpatient clinic or at completion of the recruitment period."

As well as on page 10, lines 211-213:

"Multiple cluster physicians may work within the same department or ward while being responsible for distinct patient cohorts and may therefore be randomised to different trial arms. They are asked at inclusion not to speak about the study and its intervention with colleagues."

And page 15, lines 310-317:

"Depending on the setting and workflow, tapering may be initiated during the same or a subsequent encounter, including during hospitalisation, after discharge or at a subsequent outpatient visit. During hospitalisation, the cluster physician delivers the intervention and determines the exact timing of intervention delivery. Deprescribing decisions are jointly agreed between the patient and cluster physician using the deprescribing algorithm. Where care is transferred, the communication tool conveys the decision and tapering plan to other healthcare professionals, including the GP, to support continuity of care."

<u>COMMENT 4 – REVIEWER</u>

Particularly in the hospital setting, the physician making subsequent medication changes (and thus handling deprescribing) may differ from the cluster-defining physician. e.g., if patients are enrolled on admission, the admitting senior physician acts as the cluster-defining physician, but the attending physician managing their stay and discharge actually carries out the deprescribing (and might even define a cluster in the opposing study arm). How is this handled/addressed?

<u>ANSWER 4 - REVIEWER</u>

The prescribing physicians defining the clusters are the physicians caring for the patients during the hospital stay and thus making medication changes. We clarified this aspect in the *Cluster definition* section.

Page 9, lines 193-197:

"A cluster is defined by the chief resident physician working in one of the included settings who is responsible for prescribing medication to the included patients during their hospital care or outpatient appointment at the time of enrolment. The specific level of physician responsibility is defined at each participating site according to the local organisation of care. Subsequent involvement of other physicians according to routine clinical workflows does not alter cluster allocation."

<u>COMMENT 5 – REVIEWER</u>

Does the minimum time required for physicians in their position merely serve to ensure adequate patient recruitment? should this duration not also guarantee sufficient time for the physician to perform deprescribing on "their" patients?

<u>ANSWER 5 – REVIEWER</u>

The minimum expected duration of four consecutive weeks primarily serves to ensure sufficient opportunity for patient recruitment and is not intended to ensure completion of the BZRA tapering process.

Importantly, delivery of the BE-SAFE intervention does not require deprescribing to be initiated or completed during the hospital stay. The intervention involves a patient-centred discussion and the development of an individualised deprescribing plan, including the option to defer dose reduction until after discharge or to involve the GP in initiating or continuing the plan. The agreed plan and relevant information are communicated to the patient and, where appropriate, to the GP and other healthcare professionals to support continuity of care after discharge.

Thus, the intervention can be fully delivered during the physician's period of participation even when the actual tapering occurs later.

Whether and how the agreed deprescribing plan is subsequently implemented across care transitions remains open and will be evaluated as part of the separate process evaluation.

We clarified this on page 9, lines 206-210:
"The four-week minimum is intended to ensure sufficient opportunity for patient recruitment and does not require the BZRA tapering process to be initiated or completed during the physician's period of participation, as the intervention may include a deprescribing plan to be implemented after discharge or in collaboration with the general practitioner (GP)."

Local research team and blinding

<u>COMMENT 6 – REVIEWER</u>

Local research teams: The authors mention local research teams, but their role is unclear. Please explain who recruits and who belongs to these teams in both inpatient and outpatient settings. Can local research teams "serve" clusters of both arms simultaneously? How realistic is assuming blinded local research teams when they closely collaborate with prescribing (cluster-defining) physicians who are aware of their group allocation?

<u>ANSWER 6- REVIEWER</u>

Local research teams include both blinded and unblinded staff; designated blinded staff perform patient screening, informed consent and baseline assessment before the cluster-defining physician is informed of patient inclusion and before intervention delivery. They do not collaborate with prescribing physicians, because they do not perform clinical work. Any inadvertent unblinding is documented as a protocol deviation. Unblinded staff are involved in recruitment and randomisation of cluster physicians and, hence, "serve" clusters of both arms simultaneously. Participating sites are required to have adequate research personnel and infrastructure to perform these procedures in both inpatient and outpatient settings.

We elaborated the corresponding section in the manuscript.

Page 12, lines 268-271:

"Blinded staff perform these procedures before the cluster physician is informed of the patient's inclusion and before intervention delivery (see Supplementary Figure 1)*. These staff members are not involved in clinical care and thus do not collaborate with cluster physicians."*

<u>COMMENT 7 – REVIEWER</u>

For how long? how many? (if identification is truly systematical (i.e. all(!) eligible patients identified and offered participation), the numbers of eligible patients may soon exceed planned cluster sizes...)

<u>ANSWER 7 – REVIEWER</u>

Patient screening was conducted throughout the recruitment period, but screening did not imply enrolment. The number of eligible patients at participating sites was limited, and not all

screened eligible patients ultimately met all criteria or consented to participate. Thus, exceeding the planned mean cluster size has not been an issue during recruitment, even for cluster physicians who participated during the whole study period. Furthermore, the planned cluster size is an estimation and not a fixed number to be reached and not exceeded. We clarified this last point on page 9, lines 205-206:

"Physicians are expected to remain in their position for a period sufficient to reach an estimated mean cluster size (n=8 patients), typically at least four consecutive weeks."

<u>COMMENT 8 – REVIEWER</u>

All participants receive identical study information.

Participants = members of the local research teams, or participants = cluster-defining physicians, or = patients, or all of them, or ...? and maybe consider "All ... accross both study arms receive ...". but then again, this information will probably reveal the two-arm nature of the trial, so how are "participants" prevented from deducing/guessing their cluster's allocation?

<u>ANSWER 8 – REVIEWER</u>

We clarified that "participants" refers to patients in this context. Patients receive identical study information at enrolment and are not explicitly informed of their cluster allocation. As stated in the *Blinding procedure* section, sustained patient blinding is not feasible due to the nature of the intervention.

Page 12, lines 267-274:

"All patients receive identical study information. Blinded staff perform these procedures before the cluster physician is informed of the patient's inclusion and before intervention delivery (see Supplementary Figure 1). These staff members are not involved in clinical care and thus do not collaborate with cluster physicians.

Patients are not explicitly informed of group allocation at enrolment; however, due to the nature of the intervention, sustained patient blinding is not feasible."

Screening, informed consent, enrolment and patient pathway

<u>COMMENT 9 – REVIEWER</u>

Study procedures & patient pathway: Please clarify the sequence of screening and enrolment. Are eligibility checks (including verification of 3-month BZRA use) conducted prior to patient information and consent? If so, what is the timeframe between screening and actual inclusion? At what specific point are patients actually enrolled (during outpatient consultations vs. at hospital admission)?

<u>ANSWER 9 – REVIEWER</u>

We have added a new subsection, "*Patient screening, informed consent and enrolment*", which now explicitly describes the chronological sequence and timeframes for initial screening, eligibility assessment and informed consent, baseline assessment, and enrolment. It also clarifies that patients are considered enrolled at the time of informed consent signature and

that these steps may occur during a hospital stay or across several outpatient visits, depending on the clinical pathway. To provide a more visual depiction of the patient pathway, we also added a Supplementary Figure 1.

Page 11 lines 241-252:

"Patients are identified through consecutive screening by the blinded local research team to minimise selection bias. Initial screening consists of verifying basic eligibility criteria, specifically age ≥65 years and BZRA use. These checks are performed before physician involvement and before provision of study information and formal informed consent. Patients meeting these initial criteria are subsequently assessed for all remaining inclusion and exclusion criteria, and written informed consent is obtained within 30 days of the initial screening. The baseline assessment is completed within a further 30 days after informed consent. Patients are considered enrolled at the time of informed consent signature, and time of follow-up starts at baseline assessment. Depending on the clinical pathway, these steps may take place during a single hospital stay, during an outpatient consultation, or across several outpatient visits. See Supplementary Figure 1 for more details on the patient pathway."

Intervention delivery, tapering and continuity of care

<u>COMMENT 10 – REVIEWER</u>

When is the intervention actually carried out (right at or shortly after admission, or at discharge; in the first or a subsequent outpatient visit? The intervention possibly includes tapering over a period of time; when does this happen and who is responsible?

<u>ANSWER 10 – REVIEWER</u>

The intervention is carried out anytime during hospitalisation or during a subsequent outpatient visit. It is a patient-centred shared decision-making process rather than an instruction to discontinue BZRA treatment immediately. The decision whether, when, and how to taper is made jointly with the patient and may therefore be deferred or continued after discharge. The communication tool supports continuity of the agreed plan across care transitions.

We added the following paragraph in the *Intervention* section to address these questions.
Page 15, lines 310-317:

"Depending on the setting and workflow, tapering may be initiated during the same or a subsequent encounter, including during hospitalisation, after discharge or at a subsequent outpatient visit. During hospitalisation, the cluster physician delivers the intervention and determines the exact timing of intervention delivery. Deprescribing decisions are jointly agreed between the patient and cluster physician using the deprescribing algorithm. When care is transferred, the communication tool conveys the decision and tapering plan to other healthcare professionals, including the GP, to support continuity of care."

<u>COMMENT 11 – REVIEWER</u>

Please illustrate the patient pathway through all study steps, ideally for both inpatient and outpatient settings. For instance in the inpatient setting: Who is when defined as the cluster-

defining, prescribing physician when the admitting and discharging physicians are different? Does the intervention only affect discharge medication, or is potential tapering already started during the hospital stay? Or, for an outpatient with a single visit: Is consent obtained in advance before the patient visits in person, or is there enough time for everything during one visit? Authors should try to provide a more concrete description of the practical workflows, grounded in the realities of day-to-day hospital operations.

ANSWER 11 – REVIEWER

We agree that the practical patient pathway should be clearly described. We have clarified this across the *Methods and Analysis* section, including the definition and handling of the cluster-defining physician (Comment 3), the management of subsequent prescribing and care transitions (Comment 4), the sequence of screening, informed consent, baseline assessment and enrolment (Comment 9, Supplementary Figure 1), and the timing and delivery of the intervention and subsequent tapering (Comment 10). Together, these clarifications describe the patient pathway in both inpatient and outpatient settings while allowing for variation in clinical workflows and multiple outpatient visits.

COMMENT 12 – REVIEWER

Table 2. Detailed description of BE-SAFE intervention components
Senior physician endorsement

How is this compatible with individually randomized physicians? have I missed that physicians from the same department or senior supervisor are assigned to the same study arm? but wouldn't that create nested clustering ("clusters of clusters")? and how is contamination from the senior physician to the (blinded) local research team prevented? please clarify.

ANSWER 12 – REVIEWER

We thank the reviewer for identifying a terminology issue. The cluster physician is the physician responsible for prescribing at enrolment and is the unit of randomisation; multiple cluster physicians within the same department may therefore be allocated to different arms. The "senior physician" referred to in the intervention is a senior departmental physician, who is not a cluster and is not randomised. They introduce the study to the cluster physicians but are not further involved in the intervention and do not take decisions in deprescribing. We have clarified this distinction in Table 2.

Pages 13, Table 2:
"A senior physician introduces the intervention in the department and emphasises to intervention cluster physicians that BZRA deprescribing in adults ≥65 years treated for sleep problems is a departmental priority, encourages completion of the training, and facilitates access to physician materials and patient brochures. The senior physician is not a cluster and is not randomised."

COMMENT 13 - REVIEWER

How much/far are subsequent physicians (e.g. in rehabilitation hospitals) or medical staff in nursing homes etc. involved? Are they systematically invited to continue potential tapering? This should be described in a little more detail.

ANSWER 13- REVIEWER

We clarified that the communication tool is intended to reach subsequent healthcare professionals involved in the patient's care, including GPs, rehabilitation physicians and nursing-home staff, where applicable, to support continuation of the agreed deprescribing plan across care transitions.

Page 14, Table 2:
"Structured communication forms are provided to patients/caregivers and other HCPs involved in the patient's care (e.g. GPs, rehabilitation physicians, nursing home staff), summarising discussions, patient values and preferences, decisions taken, tapering plans if applicable, and follow-up recommendations, to support continuity of care across settings."

Follow-up and participant retention

COMMENT 14 – REVIEWER

Given that patients are required to provide follow-up data for 12 months, shouldn't a willingness or capability to complete long-term follow-up be an explicit inclusion criterion? Without, I would expect high dropout rates.

ANSWER 14 – REVIEWER

We agree that willingness to participate in the 12-month follow-up is important for successful study participation. However, this was addressed as part of the informed consent process rather than as a separate eligibility criterion. The participant information provided to all potentially eligible patients clearly described the study procedures and the 12-month follow-up schedule. Patients who were unwilling or unable to participate in the required follow-up were therefore not enrolled. Capability to complete long-term follow-up cannot be guaranteed in an older patient population and was thus not included in the exclusion criteria. However, patients in end-of-life situations were excluded.

Outcomes and outcome definitions

COMMENT 15 – REVIEWER

Table 1. Population, intervention, control and outcomes
One could argue that this makes the sleep outcome a secondary outcome and having one outcome only might make things (sample size calculation etc.) easier anyway...

ANSWER 15 – REVIEWER

While we understand the argument for reclassifying the non-inferiority sleep outcome as secondary, we intentionally chose a co-primary framework utilising a sequential gatekeeping

strategy. Clinically, successful BZRA discontinuation cannot be viewed in isolation; it is only a true clinical success if it occurs without a meaningful degradation of sleep quality. We clarified this on page 16, lines 344-347:

“Sleep quality is considered co-primary because successful BZRA discontinuation should not occur at the expense of clinically meaningful deterioration in sleep. The two co-primary outcomes are therefore assessed sequentially using a gatekeeping strategy.“

COMMENT 16 – REVIEWER

Standardization of BZRA: How are different BZRAs compared (e.g., via equivalence tables)? This is relevant since dose reduction is a secondary outcome. If BZRA 1 is replaced by BZRA 2 then how is a dose reduction defined?

ANSWER 16 – REVIEWER

To standardize the BZRA metrics across different substances, we calculate the Diazepam Equivalent Dose for each participant using the conversion table of the Centre Belge d'Information Pharmacothérapeutique.

We corrected the corresponding section in Table 3 on page 17:
“Diazepam Equivalent Dose calculation via CBIP guidelines“(10)

Sample size and statistical analysis

COMMENT 17 – REVIEWER

Power & sample size: If sample sizes for two co-primary outcomes are calculated separately to yield 90% power, simply taking the maximum of the two sample sizes does not guarantee 90% power to demonstrate an effect on both outcomes. The authors do not state what overall power they aim to achieve for showing effects on both outcomes (or whether they plan to just demonstrate "at least one effect", which would be an unusual interpretation of co-primary outcomes). The authors should probably re-evaluate their sample size calculation and explicitly state underlying assumptions (crucial: co-endpoints := effect in both endpoints sought, or just in at least one?). Additionally, the actual sample sizes for both endpoints should be reported separately.

ANSWER 17 – REVIEWER

An overall study power of 80% is aimed for. This is a very important point and was indeed not mentioned in the manuscript. Since we have co-primary outcomes, both have to come up significant, which we now made clearer. With the co-primary outcomes, the overall power is indeed reduced. Assuming independence or a positive correlation, the overall power would be at least equal to the product of the individual powers, i.e. 84%. Even with a substantial negative correlation (-0.5), the overall power would stay above 80%. We now add more details about the two sample size assumptions and computations.

Page 18-19, lines 375-403:
“The sample size is based on a sequential testing procedure of the co-primary outcomes aligned with the main objectives. Superiority of the intervention for BZRA discontinuation is

tested first, followed by non-inferiority for sleep quality measured by the ISI. The trial is only considered positive if both tests are significant. […]
In order to achieve an overall power of at least 80%, we targeted a power of at least 90% for each of the co-primary outcomes. […] For the first co-primary outcome, the trial is powered to detect a relative increase of 150% in BZRA discontinuation, corresponding to discontinuation rates of 25% in the intervention group and 10% in the control group. These assumptions are informed by previous studies, with the EMPOWER trial (11) used as the primary reference due to its close comparability. A conservative intraclass correlation coefficient (ICC) of 0.05 was assumed. (11-14) Assuming a coefficient of variation of the cluster size of 0.75 and at least 62 clusters available, a mean cluster size of 5.97 patients and an overall sample size of 370 patients are required. A Pearson chi-squared test without continuity correction was used. Non-inferiority margins (second co-primary outcome) were derived from established minimally important differences and fixed at a mean difference between intervention and control of 2.5 points on the ISI. […] This resulted in a required sample size of 470 participants (approximately 235 per arm) across at least 62 clusters (mean cluster size 7.6 patients) based on a Student t-test. With this final sample size, the power for the first primary outcome increases to 93%, while maintaining the same assumptions."

COMMENT 18 – REVIEWER

Test terminology: "Superiority" is defined differently across relevant sources and regulatory guidelines. The authors should clarify their definition/understanding and also explain their somewhat unusual choice of their significance level of alpha = 2.5%.

ANSWER 18 – REVIEWER

With superiority, we refer to the superiority of either randomisation arm (i.e. a two-sided test), which is usually recommended. (15) A one-sided test at 2.5% would not cover an unlikely but not impossible superiority of the control arm (which would however not change the sample size calculations). We would therefore prefer to keep the current wording.

We clarified the choice of the alphas. As non-inferiority concerns only one direction, we consider a one-sided alpha of 2.5% for an overall type I error of 5%.
This corresponds to the conventional two-sided significance level of 5% and reflects the one-sided nature of the non-inferiority hypothesis, where only an effect in the direction of clinically relevant inferiority needs to be excluded.

Page 18, lines 382-385:

"In order to achieve an overall power of at least 80%, we targeted a power of at least 90% for each of the co-primary outcomes. The type I was set to a two-sided alpha of 0.05 and one-sided alpha of 0.025 for the co-primary outcomes, respectively. There is no inflation due to multiplicity as both outcomes have to be significant."

COMMENT 19 – REVIEWER

Please specify baseline variables; are there any beyond (standardised?) BZRA dose and sleep quality at baseline?

ANSWER 19 – REVIEWER

We added this information to the corresponding paragraph on page 20, lines 420-421:

"Baseline-adjusted analyses will be performed (16) (adjustment by age, sex, ISI and number of medications), with the adjusted analysis considered primary."

COMMENT 20 – REVIEWER

Missing data: How are (e.g., intermittent) missing values handled for participants who do not drop out?

ANSWER 20 – REVIEWER

With "drop-out" we referred to all patients with missing primary outcomes data, since the outcomes are collected at the last visit. We now omitted the term "drop-out" and only use "missing data".

Page 20, lines 443-445:

"Missing data are expected to be low. For treatment policy estimand, missing data will be ignored if ≤5% and imputed based on pre- and post-enrolment data otherwise. (17)"

COMMENT 21 – REVIEWER

Note:: test levels are denoted by alpha. If p must be is used, it should be framed as a criterion for significance (e.g., "effects will be considered significant at p < 0.025"). simply stating "p < 0.025" is inaccurate and premature, as the actual p-value will only be determined after data collection.

ANSWER 21 – REVIEWER

Thank you for having pointed this out, we now have adapted the text on page 20, lines 427-430:

"First, we will test whether the experimental group is superior to the control group with regard to BZRA discontinuation (the effect will be considered significant at p< 0.05). Only if superiority is established, we will test for non-inferiority with regard to symptom control (the effect will be considered significant at p< 0.025)."

Discussion and manuscript structure

COMMENT 22 – REVIEWER

Text/article structure: The final section of the manuscript (essentially p. 21) is repetitive, with strengths mentioned multiple times, and with a duplicate conclusion. Please shorten this as suggested in the annotated PDF, which I once again ask the authors to review.

ANSWER 22 – REVIEWER

Thank you for this suggestion. We deleted the *Potential problems and solutions* section, as well as the *Conclusions* section, as asked by the Editor. We changed the last part of the discussion, as suggested.

Page 23, lines 514-521:
"Overall, the cluster-randomised controlled BE-SAFE trial aims to generate robust evidence on the effectiveness, safety, and patient-centred impact of a multicomponent deprescribing

intervention to reduce BZRA use in older adults treated for sleep problems across diverse European healthcare settings. By evaluating BZRA discontinuation alongside non-inferiority in sleep quality and a broad range of patient-centred and safety outcomes, BE-SAFE seeks to address critical evidence gaps in this field. In the longer term, the trial aims to inform the implementation of context-sensitive, patient-centred deprescribing strategies and contribute to safer, higher-value care for the growing older population in Europe."

<u>COMMENT 23 – REVIEWER</u>

I do not understand how assessing both effectiveness and safety directly addresses tapering challenges as a key barrier to routine deprescribing. First, simply measuring an outcome does not inherently mitigate an operational barrier. Second, "tapering challenges" functions more as a restatement of "key barrier" rather than a definition or explanation of the specific nature of barrier. Third, it is not evident how evaluating effectiveness and safety will help overcome typical barriers to deprescribing like, for example, provider or patient inertia. The sentence sounds good, but does not carry too much meaning on closer inspection...

<u>ANSWER 23 – REVIEWER</u>

We appreciate this comment. We simplified the statement to its core clinical question.
Page 22, lines 485-486:

"We aim to address whether BZRA discontinuation can be achieved without compromising sleep quality."

For consistency, we also changed the wording in the *Strengths and limitations* section on page 4, lines 93-95:

"The trial is explicitly designed to address both effectiveness and clinical consequences by combining a superiority outcome (BZRA discontinuation) with a non-inferiority outcome (sleep quality) at 12 months."

Additional clarifications and minor revisions

<u>COMMENT 24 – REVIEWER</u>

Table 1. Population, intervention, control and outcomes
"consecutively recruited/included", or in what way "consecutive"?

<u>ANSWER 24 – REVIEWER</u>

In this context, *consecutive* means that we approached every single eligible patient who met the criteria in the exact order they appeared on screening lists, without skipping any or hand-picking participants.

<u>COMMENT 25 – REVIEWER</u>
Table 1. Population, intervention, control and outcomes

Consider clarifying: last 3 months prior to study invitation or prior to hospitalization etc.?

ANSWER 25 – REVIEWER

We added "*prior to study invitation*".

Page 8, Table 1:
"Patient level: Consecutive older adults aged ≥65 years using BZRA on average ≥3 times per week for at least the last 3 months prior study invitation, prescribed for sleep problems."

COMMENT 26 - AUTHOR'S ADDITIONAL CLARIFICATION

Following further discussion within the study team, we revised the Statistical Analysis Plan (SAP) to prespecify exploratory analyses of intervention effect heterogeneity by country, in addition to patient-level characteristics. The corresponding wording has also been updated in the manuscript.

Page 20, lines 438-440:

"Prespecified exploratory analyses will assess intervention effect heterogeneity by introducing interaction terms into the primary analysis. These analyses will be limited to country and patient-level characteristics in the full analysis set (FAS), following the intention-to-treat principle, and will be considered exploratory, without adjustment for multiple testing."

The revised SAP has been updated accordingly.

COMMENT 27 - AUTHOR'S ADDITIONAL CLARIFICATION

The Data Availability Statement has been clarified to reflect that this manuscript reports the study protocol and that no datasets have yet been generated or analysed for the present protocol manuscript.

On page 26, lines 581-583, the statement now reads:

"Data sharing not applicable as no datasets generated and/or analysed for this study. No datasets have yet been generated or analysed for the present protocol manuscript."

References

(1) Association WM. World Medical Association Declaration of Helsinki: Ethical Principles for Medical Research Involving Human Subjects. *JAMA* 2013;310(20):2191–94. doi: 10.1001/jama.2013.281053

(2) International Council for Harmonisation of Technical Requirements for Pharmaceuticals for Human Use. ICH E6 (R2) Guideline for Good Clinical Practice. Geneva, Switzerland, 2016.

(3) Shapoval V, de Saint Hubert M, Pétein C, et al. Barriers to discontinuing benzodiazepine receptor agonists in older adults: a survey of older adults across Europe. *Age Ageing* 2025;54(12) doi: 10.1093/ageing/afaf342

(4) Shapoval V, Evrard P, Sibille FX, et al. Barriers to and Enablers of Supporting Deprescribing Benzodiazepines in Older Adults: A Survey of European Nonphysician Healthcare Professionals. *Basic Clin Pharmacol Toxicol* 2025;137(4):e70100. doi: 10.1111/bcpt.70100

(5) Shapoval V, de Saint Hubert M, Evrard P, et al. Barriers to Deprescribing Benzodiazepines in Older Adults in a Survey of European Physicians. *JAMA Network Open* 2025;8

(6) Zeraatkar D, Nagraj SK, Ling M, et al. Comparative effectiveness of interventions to facilitate deprescription of benzodiazepines and other sedative hypnotics: systematic review and meta-analysis. *BMJ* 2025;389:e081336. doi: 10.1136/bmj-2024-081336

(7) López-Toribio M, Dalleur O, Adda TR, et al. Mapping care trajectories for hospital-initiated benzodiazepine deprescribing in older adults: a multicentre qualitative study. *BMC Health Services Research* 2025;25(1):1578. doi: 10.1186/s12913-025-13725-2

(8) Agoritsas T, Kumbargere Nagraj S, Callegari E, et al. Deprescription of benzodiazepine and sedative hypnotics in insomnia disorder: a clinical practice guideline. BE-SAFE Deprescription recommendations. MAGICapp. 2023.

(9) Sibille FX, Salbert J, Bolt L, et al. Equipping physicians for benzodiazepine receptor agonists deprescription in older adults: theory-based development of the BE-SAFE intervention. *Res Social Adm Pharm* 2025;21(9):714–21. doi: 10.1016/j.sapharm.2025.05.002 [published Online First: 20250521]

(10) Belgian Centre for Pharmacotherapeutic Information (CBIP). Benzodiazépines – conversion des benzodiazépines et Z-drugs (dose équivalente). 2026. Available from: https://www.cbip.be/fr/chapters/11?frag=7476 [cited 2026 Aug 20].

(11) Tannenbaum C, Martin P, Tamblyn R, et al. Reduction of inappropriate benzodiazepine prescriptions among older adults through direct patient education: the EMPOWER cluster randomized trial. *JAMA Intern Med* 2014;174(6):890–8. doi: 10.1001/jamainternmed.2014.949

(12) Adam L, Moutzouri E, Baumgartner C, et al. Rationale and design of OPtimising thERapy to prevent Avoidable hospital admissions in Multimorbid older people (OPERAM): a cluster randomised controlled trial. *BMJ Open* 2019;9(6):e026769. doi: 10.1136/bmjopen-2018-026769 [published Online First: 20190603]

(13) Blum MR, Sallevelt B, Spinewine A, et al. Optimizing Therapy to Prevent Avoidable Hospital Admissions in Multimorbid Older Adults (OPERAM): cluster randomised controlled trial. *Bmj* 2021;374:n1585. doi: 10.1136/bmj.n1585 [published Online First: 20210713]

(14) Health Services Research Unit UoA. Empirical estimates of ICCs from changing professional practice studies. Aberdeen, UK: Health Services Research Unit, University of Aberdeen, 2020.

(15) International Conference on Harmonisation (ICH). ICH harmonised tripartite guideline: statistical principles for clinical trials E9. Current Step 4 version. 1998. Available: ich.org [Accessed 22 Aug 2026]

(16) Yang S, Starks MA, Hernandez AF, et al. Impact of baseline covariate imbalance on bias in treatment effect estimation in cluster randomized trials: Race as an example. *Contemp Clin Trials* 2020;88:105775. doi: 10.1016/j.cct.2019.04.016 [published Online First: 20190620]

(17) Little RJ, D'Agostino R, Cohen ML, et al. The prevention and treatment of missing data in clinical trials. N Engl J Med 2012;367(14):1355–60. doi: 10.1056/NEJMsr1203730
