# Supplementary material for: Implementing a patient-centred and evidence-based intervention to reduce BEnzodiazepine and sedative-hypnotic use to improve patient SAFEty and quality of care (BE-SAFE): study protocol of a cluster randomised controlled trial
Source: BMJ Open. 2026 Oct 12;16(10):e123804. doi: 10.1136/bmjopen-2026-123804 (PMC13662039; doi:10.1136/bmjopen-2026-123804)
Supplement: Supplementary file 3 [file bmjopen-16-10-s001.docx]

**Initial screening (blinded)**

Verify basic eligibility criteria:

- Age ≥ 65
- BZRA use

**Intervention delivery (within 5 days) or usual care**

**Full eligibility assessment (blinded)**

Verify all remaining inclusion and exclusion criteria

**Informed consent**

**Baseline assessment**

**Enrolment**

Patients are considered enrolled at the time of informed consent signature. Time of follow-up starts at baseline.

**Cluster physician informed of patient inclusion**

Not meeting basic criteria= not eligible

Not meeting remaining criteria= not eligible

within 30 days

within 30 days

Supplementary Figure 1. Patient pathway from screening to intervention delivery

The time windows apply for inpatient and outpatient settings. The whole process may take place during one single day or over several days/outpatient visits.
