# Supplementary material for: Implementing a patient-centred and evidence-based intervention to reduce BEnzodiazepine and sedative-hypnotic use to improve patient SAFEty and quality of care (BE-SAFE): study protocol of a cluster randomised controlled trial
Source: BMJ Open. 2026 Oct 12;16(10):e123804. doi: 10.1136/bmjopen-2026-123804 (PMC13662039; doi:10.1136/bmjopen-2026-123804)
Supplement: Supplementary file 5 [file bmjopen-16-10-s003.docx]

SUPPLEMENTARY FILE 2

Ethics approvals by participating country and institution

| Country | Ethics Committee | Approval number |
| --- | --- | --- |
| Belgium | Comité d’éthique CHU UCL Namur, site Godinne | NUB0392024000049 – 111/2024 |
|  | Comité d’éthique CHU UCL Namur, site St. Elisabeth | NUB0392024000049 – 19/2024 |
|  | Comité d’éthique CHU UCL Namur, site Dinant | NUB0392024000049 – 5/2025 |
| Poland | Bioethics Committee of the Institute of Psychiatry and Neurology | 10/2024 |
| Norway | Regional Committee for Medical and Health Research Ethics | REK sør-øst C - 722845 |
| Greece | Ethics Committee of Eginition Hospital | 1217/02-08-2024 |
|  | Ethics Committee of Laikon Hospital | 606/03-09-2024 |
|  | Ethics Committee of Sotiria Hospital | 3532/10-02-2026 |
|  | Ethics Committee of Ippokratio Hospital | 65/16-07-202 |
| Spain | The Commission on Ethics in Animal and Human Experimentation (CEEAH) of the Universitat Autònoma de Barcelona | 6221 |
|  | Ethics Committee of Germans Trias I Pujol Hospital of Badalona | PI-24-206 |
| Switzerland | The Ethical Committee of the Canton of Bern | BASEC2024-00702 |
